# Relationship between intraventricular mechanical dyssynchrony and left ventricular systolic and diastolic performance: An in vivo experimental study

**DOI:** 10.14814/phy2.15607

**Published:** 2023-02-17

**Authors:** Manuel Ignacio Monge García, Zhongping Jian, Feras Hatib, Jos J. Settles, Maurizio Cecconi, Michael R. Pinsky

**Affiliations:** ^1^ Intensive Care Unit Hospital Universitario SAS Jerez Jerez de la Frontera Spain; ^2^ Edwards Lifesciences Irvine California USA; ^3^ Department Anaesthesia and Intensive Care Units, Humanitas Research Hospital Humanitas University Milan Italy; ^4^ Department of Critical Care Medicine University of Pittsburgh School of Medicine Pittsburgh Pennsylvania USA

**Keywords:** diastolic function, mechanical dyssynchrony, myocardial energetics, pressure–volume loop, ventriculo‐arterial coupling

## Abstract

Left ventricular mechanical dyssynchrony (LVMD) refers to the nonuniformity in mechanical contraction and relaxation timing in different ventricular segments. We aimed to determine the relationship between LVMD and LV performance, as assessed by ventriculo‐arterial coupling (VAC), LV mechanical efficiency (LV_eff_), left ventricular ejection fraction (LVEF), and diastolic function during sequential experimental changes in loading and contractile conditions. Thirteen Yorkshire pigs submitted to three consecutive stages with two opposite interventions each: changes in afterload (phenylephrine/nitroprusside), preload (bleeding/reinfusion and fluid bolus), and contractility (esmolol/dobutamine). LV pressure–volume data were obtained with a conductance catheter. Segmental mechanical dyssynchrony was assessed by global, systolic, and diastolic dyssynchrony (DYS) and internal flow fraction (IFF). Late systolic LVMD was related to an impaired VAC, LV_eff_, and LVEF, whereas diastolic LVMD was associated with delayed LV relaxation (logistic tau), decreased LV peak filling rate, and increased atrial contribution to LV filling. The hemodynamic factors related to LVMD were contractility, afterload, and heart rate. However, the relationship between these factors differed throughout the cardiac cycle. LVMD plays a significant role in LV systolic and diastolic performance and is associated with hemodynamic factors and intraventricular conduction.

## Graphical abstract

Hemodynamic and electrical factors associated with left ventricular mechanical dyssynchrony during the cardiac cycle and their relationship with the systolic and diastolic ventricular function.

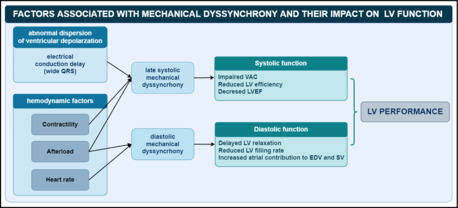

## 1 INTRODUCTION

The term synchronism is often used to refer to the ability of a system to coordinate different events and operate in unison. Left ventricular mechanical dyssynchrony (LVMD), therefore, refers to the nonuniformity in mechanical contraction and relaxation between different ventricular segments (Brutsaert, 1987; de Souza Leite & Rassier, 2020; Fudim, Dalgaard, et al., 2021). Nonuniformity is a physiological characteristic of the cardiac pump related to the complex ventricular geometry, the nonuniform distribution of the electrical impulse, and the heterogeneous activation‐inactivation pattern of the sarcomeres (Brutsaert, 1987; de Souza Leite & Rassier, 2020). Although a minimal degree of mechanical dyssynchrony seems to be a typical feature of normal cardiac physiology (Brutsaert, 1987; Haendchen et al., 1983), it may be aggravated by pathological conditions such as heart failure (Yu et al., 2007), hypertrophic myocardiopathy (Nagakura et al., 2007; Tsai et al., 2018), arterial hypertension (Sun et al., 2015), or coronary artery disease‐induced regional myocardial ischemia (Fudim, Fathallah, et al., 2019; Lee et al., 2011).

Abnormal intraventricular conduction manifested as widening the QRS complex, as in bundle branch block (Revah et al., 2016; Sillanmaki et al., 2020) or during artificial ventricular pacing (Johnson et al., 2009; Liakopoulos et al., 2006), also leads to increased mechanical dyssynchrony. However, while QRS duration reflects intraventricular electrical delay, LVMD quantifies regional mechanical events. Both phenomena, however, are not necessarily related. LVMD may be present without anomalous electrical conduction (Ghio et al., 2004; Yu et al., 2003) and can be affected by non‐electrical factors (Gwirtz et al., 1986; Kim et al., 2011; Kurita et al., 2007; Miura et al., 1993). Moreover, mechanical rather than electric synchronization seems more relevant (Breithardt et al., 2002; Steendijk et al., 2006) and has been identified as an independent predictor of the long‐term follow‐up of patients with chronic heart failure or coronary artery disease, even in the absence of an abnormal electrical activation (Bader et al., 2004; Cho et al., 2005; Hess et al., 2017).

Since increased LVMD reduces ventricular ejection efficiency (Park et al., 2007; Schreuder et al., 2005) and LV diastolic function (Aoyagi et al., 1993), we hypothesize that LVMD‐induced reduced LV performance would have both systolic and diastolic LVMD characteristics that may induce different effects on LV ventricular function. So, we aimed to quantify the association between LV mechanical dyssynchrony and LV systolic and diastolic function during experimental changes in loading and contractile conditions in an intact cardiovascular system and determine the factors modulating this relationship.

## 2 METHODS

### 2.1 Anesthesia and surgical preparation

Experiments were performed in 13 Yorkshire crossbred female domestic pigs (*Sus scrofa domesticus*) of 5.6 ± 0.7 (mean ± SD) months old, weighing 82 ± 5 kg. Animals were maintained in a temperature and humidity‐controlled rooms with a 12‐hour light–dark cycle and standard chow and tap water ad libitum. The animals were premedicated with an intramuscular combination of telazol (4.4 mg kg^−1^), ketamine (2.2 mg kg^−1^), and xylazine (1.1 mg kg^−1^). They were orally intubated, and their lungs were mechanically ventilated in volume‐controlled mode using a fraction of inspired oxygen of 0.6, an inspiratory to expiratory ratio of 1:2.5, tidal volume of 10 mL/kg plus 100 mL compensation for dead space, and a respiratory rate adjusted to maintain a PaCO_2_ of between 4.5 and 6 kPa. General anesthesia was maintained by 1%–2% isoflurane and a mixture of oxygen, air, and/or nitrous oxide without a neuromuscular blocking agent. Fluid maintenance was provided by an intravenous infusion of Ringer's lactate solution (4 mL kg^−1^ h^−1^). Rectal temperature was monitored and kept between 38 and 40°C using a heating pad. The depth of anesthesia was evaluated every 15 min by inspecting the jaw tone and the toe pinch response. Animals were sacrificed at the end of the study with a lethal dose of sodium pentobarbital (90 mg kg^−1^).

### 2.2 Data collection and analysis

Instantaneous LV pressure–volume measurements were obtained from a 7Fr‐lumen dual‐field conductance catheter with 12 equidistant electrodes and a high‐fidelity pressure sensor between the 5th and 6th electrodes (CA71083‐PL; CD Leycom), and connected to a pressure–volume signal processor (Inca®; CD Leycom). The catheter was inserted through the left external carotid artery and positioned along the LV long axis. Correct placement was confirmed by fluoroscopy and the inspection of the morphology of the individual segmental LV pressure–volume loops. In the swine heart, five LV segments defined for each pair of electrodes from third to eighth typically cover the LV cavity from base to apex. The remaining electrodes were excluded for the pressure–volume analysis. A fluid‐filled pulmonary artery catheter (Swan Ganz CCOmbo 777HF8; Edwards Lifesciences) was inserted percutaneously through the right internal jugular vein to the pulmonary artery.

The volume signal was calibrated using the thermodilution method via right‐heart catheterization. The average value of 3–5 thermodilution ice‐cold saline boluses randomly injected during the respiratory cycle was used to determine cardiac output (CO). Correction for parallel conductance (the conductance of the surrounding tissues, which was subtracted from the raw catheter signal) was performed with 10 mL boluses of 5% hypertonic saline through the distal port of the pulmonary artery catheter (Baan et al., 1984). The conductance signals were then converted to calibrated volume signals by considering the inter‐electrode spacing, the parallel conductance correction, and the CO calibration factor obtained from thermodilution. CO calibration and parallel conductance correction were performed before the experimental protocol and repeated after fluid volume intervention. The signals were recorded at 250 Hz and filtered using a 25 Hz low‐pass filter. Before and after each experimental stage, a transient occlusion of the inferior vena cava (IVC) using a 25 mm Fogarty balloon (Edwards Transfemoral Balloon Catheter, 9350 BC25; Edwards Lifesciences) was performed. The ventilator was transiently turned off during all measurements. This procedure was repeated if ectopic beats or no significant decrease in LV pressure was detected. Radial arterial pressure was continuously monitored with a fluid‐filled pressure transducer (TruWave DPT; Edwards Lifesciences), leveled with the heart, and zeroed at the beginning of the study. The radial pressure waveform was recorded at a sampling rate of 100 Hz using the EV1000 monitor (Edwards Lifesciences) and filtered to remove noise and artifacts using a 10 Hz low‐pass filter. The QRS duration was determined by interpolating the intracardiac ECG signal to 1 kHz and decomposing it using the maximal overlap discrete wavelet transform with a Sym4 wavelet to extract the Q, R, and S features (Figure S1). Pressure–volume and dyssynchrony analysis were performed with a custom‐made software.

### 2.3 Global cardiac function

End‐systolic pressure (Pes), stroke volume (SV), CO, LV end‐diastolic volume (EDV), LV end‐systolic volume (ESV), LV end‐diastolic pressure (EDP), and LVEF calculated from five beats in steady‐state conditions during a respiratory pause before the IVC occlusion. Effective arterial elastance (Ea = Pes/SV) was used as a lumped parameter of LV afterload (Sunagawa et al., 1984). LV end‐systolic elastance (Ees) was calculated using the iterative regression method (Kass et al., 1986). The slope of the end‐systolic pressure–volume relationship (ESPVR) was calculated from the linear regression analysis of the maximal elastance (E) points on each cardiac cycle, defined as *E*(*t*) = *P*(*t*)/*V*(*t*) − *V*_0_, where *V*_0_ is volume‐axis intercept or the LV unstressed volume. End‐systole was defined at the maximum value of the LV elastance (Kono et al., 1984).

### 2.4 Assessment of LV diastolic function

We calculated the LV chamber stiffness from the exponential curve fit of the end‐diastolic pressure–volume relationship (EDPVR) as $\mathrm{EDP}=\alpha \times e^{\beta \times \mathrm{EDV}}$, being *α* the curve fitting constant and *β* the LV stiffness constant (Mirsky, 1984). LV relaxation time constant (*τ*) was calculated using the logistic method (Matsubara et al., 1995; Ogilvie et al., 2020). The end of isovolumetric relaxation was defined when LV pressure returned to the level of preceding EDP+5 mmHg (Matsubara et al., 1995). Peak filling rate (PFR) was calculated as the maximum value of the first derivate of LV volume. The peak of the R wave on the ECG was used for defining the end of diastole.

### 2.5 Assessment of ventriculo‐arterial coupling and left ventricular efficiency

Ventriculo‐arterial coupling (VAC) was defined as the ratio of Ea to Ees (Sunagawa et al., 1983). LV pressure–volume area (PVA) characterizes the total mechanical energy and was determined as the sum of stroke work (SW) and potential energy (PE), where SW is the area within the PV loop and PE = 0.5 × Pes × (ESV − *V*_0_). The ratio of SW/PVA represents the LV mechanical efficiency (LV_eff_) of transferring the total mechanical energy into SW (Asanoi et al., 1989; Burkhoff & Sagawa, 1986).

### 2.6 Left atrial function assessment

The onset of the atrial systole was determined by analyzing the LV volume curve at the point where the ventricular volume increases before end‐diastole, which usually occurs at the last zero‐crossing of the first derivative of LV volume (Figure S2). Effective atrial stroke volume (SV_atria_), which does not consider the loss in pulmonary veins, was calculated by subtracting the LV volume at the onset of atrial systole to EDV. Similarly, the atrial systolic pressure gradient was calculated as the difference between EDP and the pressure at the beginning of atrial systole. Atrial power was determined as the product of SV_atria_ and atrial systolic pressure gradient divided by the duration of atrial systole, expressed in watts. The contribution of the atrial contraction to the LV preload and SV was calculated as SV_atria_/EDV and SV_atria_/SV, respectively, and expressed as a percentage.

### 2.7 Assessment of LV intraventricular mechanical dyssynchrony

**FIGURE 1:**
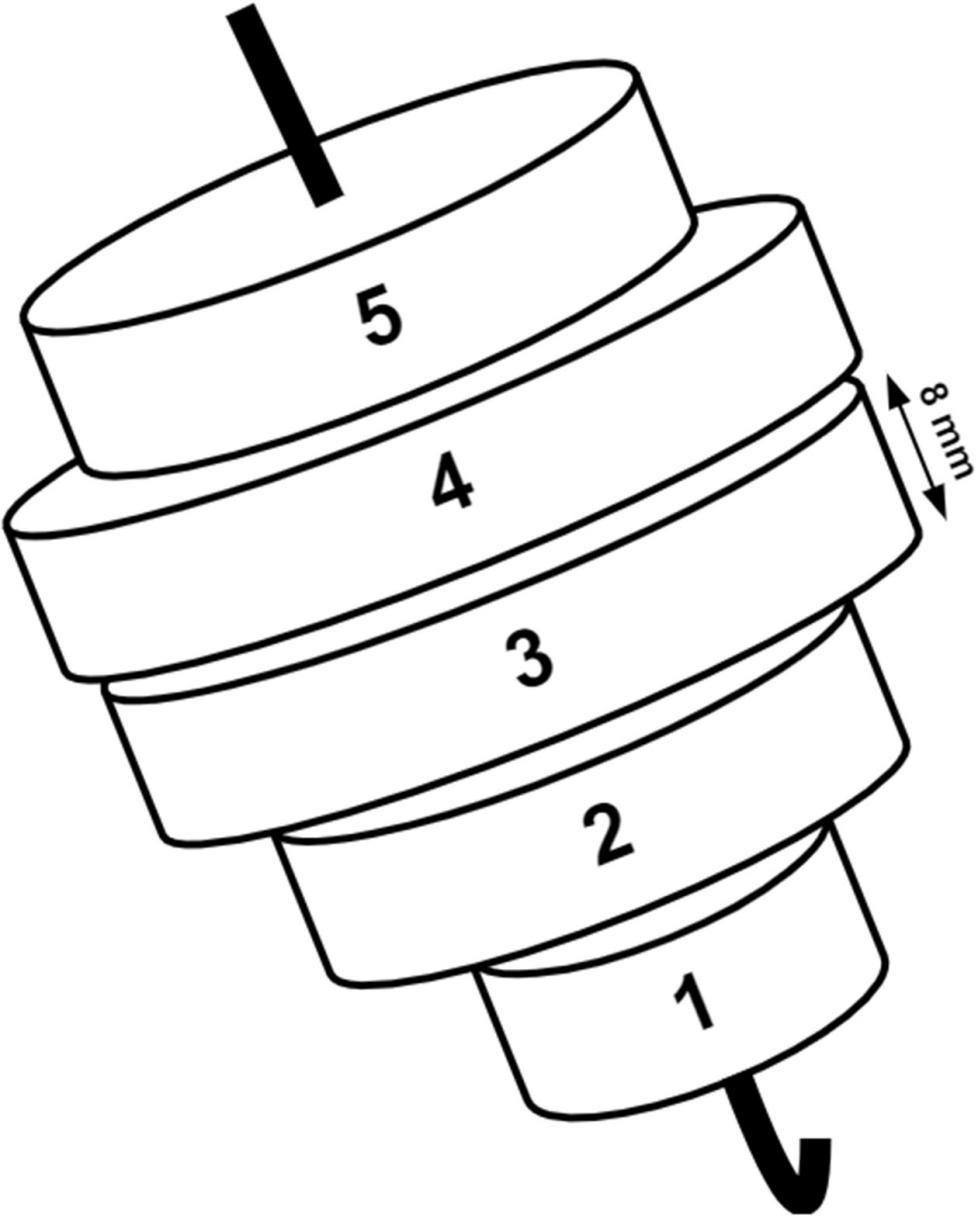
Schematic segmentation of the left ventricle. Each ventricular segmental volume was defined by two electrodes separated by 8 mm used to calculate the electrical conductance by the measuring electrodes.

LV dyssynchrony was assessed by the LV global mechanical dyssynchrony (DYS) and the internal flow fraction (IFF) from the ventricular segmental volumes (*V*_seg_) obtained from the conductance catheter (Steendijk et al., 2004). The conductance‐derived segmental volumes reflect the instantaneous volume slices perpendicular to the LV long axis (segment one being closer to the apex, Figure 1) as obtained by cine‐computerized tomography (van der Velde et al., 1992). A segment is considered dyssynchronous when the segmental volume changes opposite the total LV volume change. Segmental dyssynchrony was quantified as the percentage of the cardiac cycle that a ventricular segment is dyssynchronous. Global LV dyssynchrony (DYS) was calculated as the average of the five segmental dyssynchrony values. Conductance catheter‐derived indexes of LVMD strongly correlated with sept al‐to‐lateral dyssynchrony indexes derived from tissue‐Doppler echocardiographic measurements (Kurita et al., 2007; Steendijk et al., 2004).

Internal flow (IF) quantifies the segment‐to‐segment blood volume shift produced by nonuniform contraction and filling, which results in an ineffective filling or ejection (Steendijk et al., 2004). IF was calculated as the difference between the sum of the absolute segmental volume (*V*_seg_) changes and the absolute total volume (*V*_total_) change:

$$\text{IF}\left(t\right)=\left(\sum_{i=1}^{5}\left|{\mathrm{dV}}_{\mathrm{seg},i}\left(t\right)/\mathrm{dt}\right|-\left|{\mathrm{dV}}_{\text{total}}\left(t\right)/\mathrm{dt}\right|\right)/2.$$

Internal flow fraction (IFF) was calculated by integrating IF over the cardiac cycle duration (*T*) and dividing by the absolute effective flow time integral:

$$\mathrm{IFF}=\int_{0}^{T}\text{IF}/\int_{0}^{T}\left|{\mathrm{dV}}_{\text{total}}\left(t\right)/\mathrm{dt}\right|.$$

**FIGURE 2:**
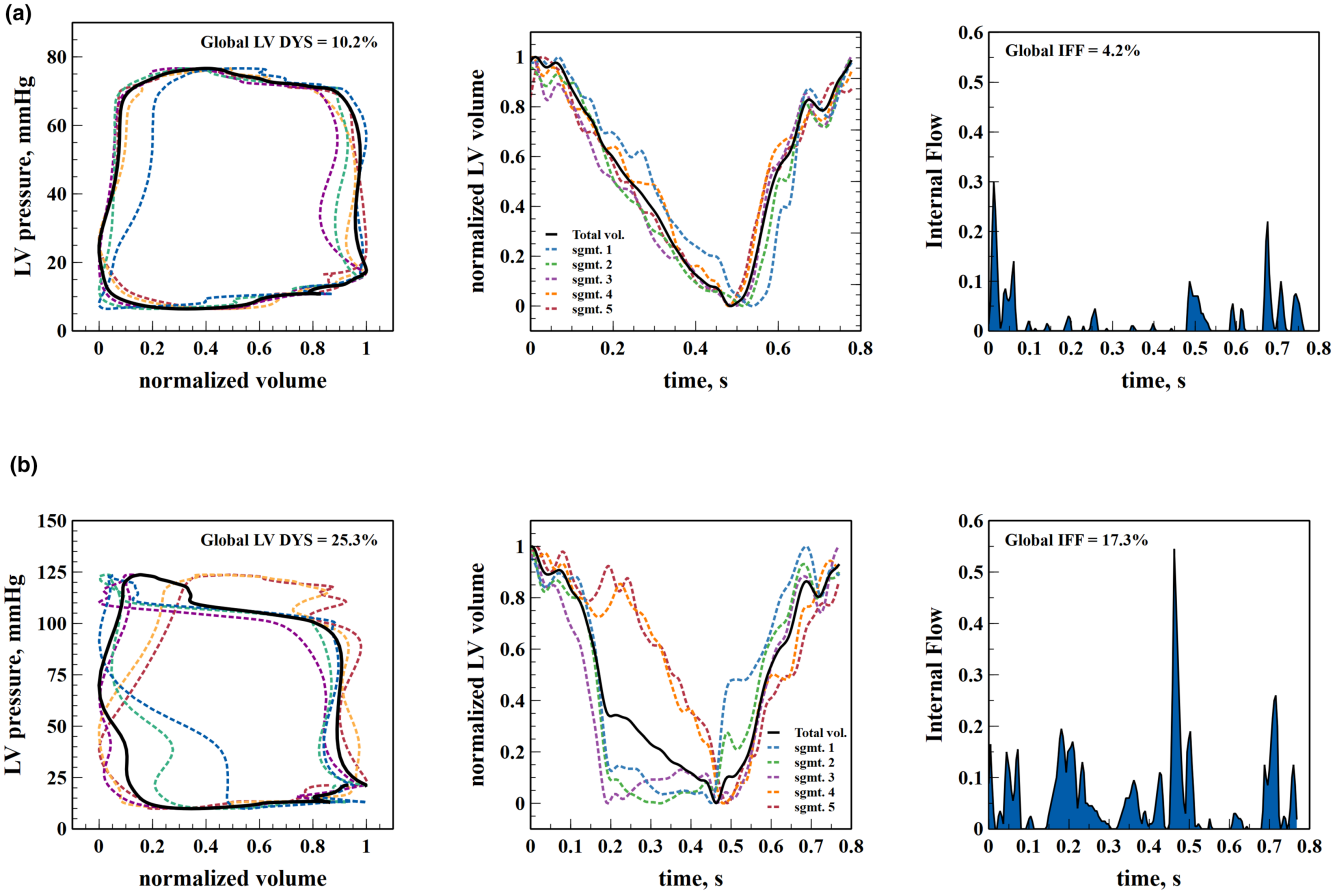
Representative example of the global and segmental pressure–volume loops, global left ventricular and segmental time–volume curves, and internal flow in the same experimental animal before and after phenylephrine infusion (a and b, respectively). Total and segmental volumes were normalized rescaling the data range to [0,1] for a better comparison of the morphological and time discrepancies in pressure–volume loops and time–volume curves. Note the distorted segmental pressure–volume loops out of sync after phenylephrine infusion.

Global DYS and IFF were calculated as the mean of the overall segmental dyssnchronies for each cardiac cycle and averaged over five consecutive beats [median: 5 beats (25th to 75th interquartile: 4 to 7 beats)] (Figure 2). DYS and IFF were also divided into their systolic and diastolic components. The systole was subdivided into early (from R wave to *dP*/*dt*_max_) and late systole (from *dP*/*dt*_max_ to Emax), and the diastole into early (from Emax to PFR) and late diastole (from PFR to R wave) (Schreuder et al., 2005). For each specified interval, mechanical dyssynchrony was quantified as the percentage of the cardiac cycle interval that *V*_seg_ was dyssynchronous.

### 2.8 Experimental protocol

Animals were initially resuscitated with colloid boluses (Voluven®, 130/0.4; Fresenius Kabi Deutschland GmbH) until there was no further increase in CO. Then, they were allowed to stabilize for at least 10 min (heart rate and MAP variation <5%). The study protocol consisted of three consecutive stages with two opposite interventions: changes in afterload (phenylephrine and sodium nitroprusside), preload (bleeding and fluid bolus), and contractility (esmolol and dobutamine). The experiment started with the afterload interventions: animals received a phenylephrine infusion (30–120 mg kg^−1^ min^−1^) to increase MAP by 40% mmHg from baseline, and then, they were allowed to recover. Then, they were treated with sodium nitroprusside (100–200 mg kg^−1^ min^−1^) to decrease MAP by 40% from baseline but not below 50 mmHg to allow an adequate hemodynamic tolerance during the IVC occlusions, followed by recovery to baseline status. Subsequently, for preload interventions, the animals were submitted to stepwise bleeding of 12 mL kg^−1^ (50 mL min^−1^), and the blood was stored in a heparinized sterile bag. Then, the blood was reinfused at the same rate, and an additional fluid bolus of 10 mL kg^−1^ of a colloid was infused in 5 min. After the fluid administration, the contractility interventions followed: an esmolol infusion was started at 50 μg kg^−1^ min^−1^ and increased until reducing LV *dP*/*dt*_max_ by 50% from its previous value, with a limit dose of 200 μg kg^−1^ min^−1^. Then, the esmolol infusion was stopped and, after a recovery period, a dobutamine infusion (5 μg kg^−1^ min^−1^) was used to increase LV *dP*/*dt*_max_ by >50%. LV PV loops and arterial pressure waveforms were obtained at each baseline and after each intervention. Animals were euthanized at the end of the study with an intravenous dose of sodium pentobarbital (90 mg kg^−1^).

### 2.9 Statistical analysis

Data are expressed as means ± standard deviation. Differences during interventions were assessed by a paired *t*‐test. The coefficient of determination was calculated as a marker of the goodness of fit for the ESPVR and EDPVR. The relationship between continuous variables was assessed by a generalized linear mixed‐effects model analysis with the restricted maximum likelihood (REML) and the Kenward‐Roger degrees of freedom adjustment, using individual animals as a subject for random factors and experimental stages as repeated measurements. A *p*‐value <0.05 was considered statistically significant. The authors had full access to and took full responsibility for the integrity of the data. All authors have read and agreed to the manuscript as written.

## 3 RESULTS

### 3.1 Global hemodynamics

A detailed description of the hemodynamic variables during afterload, preload, and contractility interventions is shown in Tables S1, S2, and S3, respectively. Dyssynchrony and IFF during different hemodynamic interventions are shown in Table 1. Phenylephrine increased MAP by 42 ± 9% and LV ESP by 45 ± 10%, while sodium nitroprusside decreased by 35 ± 6% and 35 ± 6%, respectively. During changes in preload, bleeding reduced EDV by 10 ± 8%, and reinfusion plus fluid administration increased by 29 ± 21%. Neither CO nor SV were significantly modified after bleeding but increased after fluid bolus (22 ± 20%, and 21 ± 30%, respectively). During contractility interventions, esmolol decreased LV *dPdt*_max_ by 46 ± 18%, while dobutamine increased by 88 ± 22%.

**TABLE 1:** Left ventricular mechanical dyssynchrony (DYS) and internal flow fraction (IFF) during different experimental conditions (*n* = 13).

	Phenylephrine		*p*‐value*	Sodium nitroprusside		*p*‐value*
	Before	After		Before	After	
Global DYS, %	14 ± 2	16 ± 4	0.031	14 ± 4	14 ± 3	0.996
Systolic DYS	8 ± 3	12 ± 6	0.005	10 ± 6	9 ± 5	0.008
Diastolic DYS	18 ± 3	19 ± 3	0.340	18 ± 3	19 ± 3	0.250
Global IFF, %	6 ± 2	9 ± 3	<0.001	7 ± 3	6 ± 3	0.063
Systolic IFF	3 ± 1	7 ± 5	0.003	5 ± 4	4 ± 4	0.086
Diastolic IFF	8 ± 3	11 ± 3	<0.001	9 ± 3	8 ± 3	0.141

	Bleeding		*p*‐value*	Reinfusion + fluid loading		*p*‐value*
	Before	After		Before	After	
Global DYS, %	15 ± 4	15 ± 3	0.316	15 ± 2	13 ± 3	0.075
Systolic DYS	11 ± 6	10 ± 5	0.319	10 ± 5	9 ± 5	0.365
Diastolic DYS	18 ± 3	19 ± 3	0.256	18 ± 3	17 ± 3	0.155
Global IFF, %	7 ± 3	7 ± 3	0.962	7 ± 3	6 ± 3	0.213
Systolic IFF	6 ± 4	5 ± 3	0.678	5 ± 4	4 ± 3	0.253
Diastolic IFF	9 ± 3	9 ± 3	0.755	9 ± 3	8 ± 3	0.239

	Esmolol		*p*‐value*	Dobutamine		*p*‐value*
	Before	After		Before	After	
Global DYS, %	13 ± 3	16 ± 2	<0.001	14 ± 3	13 ± 3	0.112
Systolic DYS	9 ± 4	11 ± 3	0.009	9 ± 5	9 ± 5	0.746
Diastolic DYS	17 ± 3	20 ± 2	0.002	18 ± 3	16 ± 4	0.125
Global IFF, %	6 ± 3	9 ± 3	<0.001	7 ± 3	5 ± 2	0.116
Systolic IFF	4 ± 3	6 ± 4	0.003	4 ± 4	4 ± 2	0.886
Diastolic IFF	8 ± 4	10 ± 3	0.004	9 ± 3	7 ± 5	0.047

*Note*: Values are presented as means ± SD.

Abbreviations: DYS, intraventricular mechanical dyssynchrony; IFF, internal flow fraction.

* Paired *t*‐test for before and after each experimental intervention.

### 3.2 Changes in VAC, LV_eff_ , and diastolic function

The median overall coefficient of determination for the ESPVR fit was 0.975 (95% confidence interval: 0.972 to 0.977) and 0.969 (95% CI: 0.963 to 0.975) for the EDPVR. Whereas phenylephrine increased VAC because of a more noticeable increase in Ea, nitroprusside and bleeding improved VAC by a selective decrease in Ea. Fluid loading increased VAC by reducing Ees, whereas esmolol impaired VAC by increasing Ea without any significant change in Ees. However, the ESV increased with esmolol, reflecting a rightward shift of the LV ESPVR. Dobutamine improved VAC by enhancing Ees without changes in Ea. LV_eff_ decreased with phenylephrine (relative decrease: 8 ± 8%), fluid bolus (11 ± 10%), and esmolol (27 ± 14%), and it improved with nitroprusside (relative increase: 15 ± 14%), bleeding (14 ± 11%), and dobutamine (21 ± 10%). LV relaxation (*τ*) was delayed by 4 ± 2 ms with phenylephrine and 6 ± 4 ms with esmolol and reduced with dobutamine by 3 ± 2 ms. Preload changes did not affect LV relaxation. LV stiffness constant (*β*) decreased with bleeding and increased with fluid loading and esmolol (Tables S1–S3).

### 3.3 Distribution and changes in LV intraventricular mechanical dyssynchrony

**FIGURE 3:**
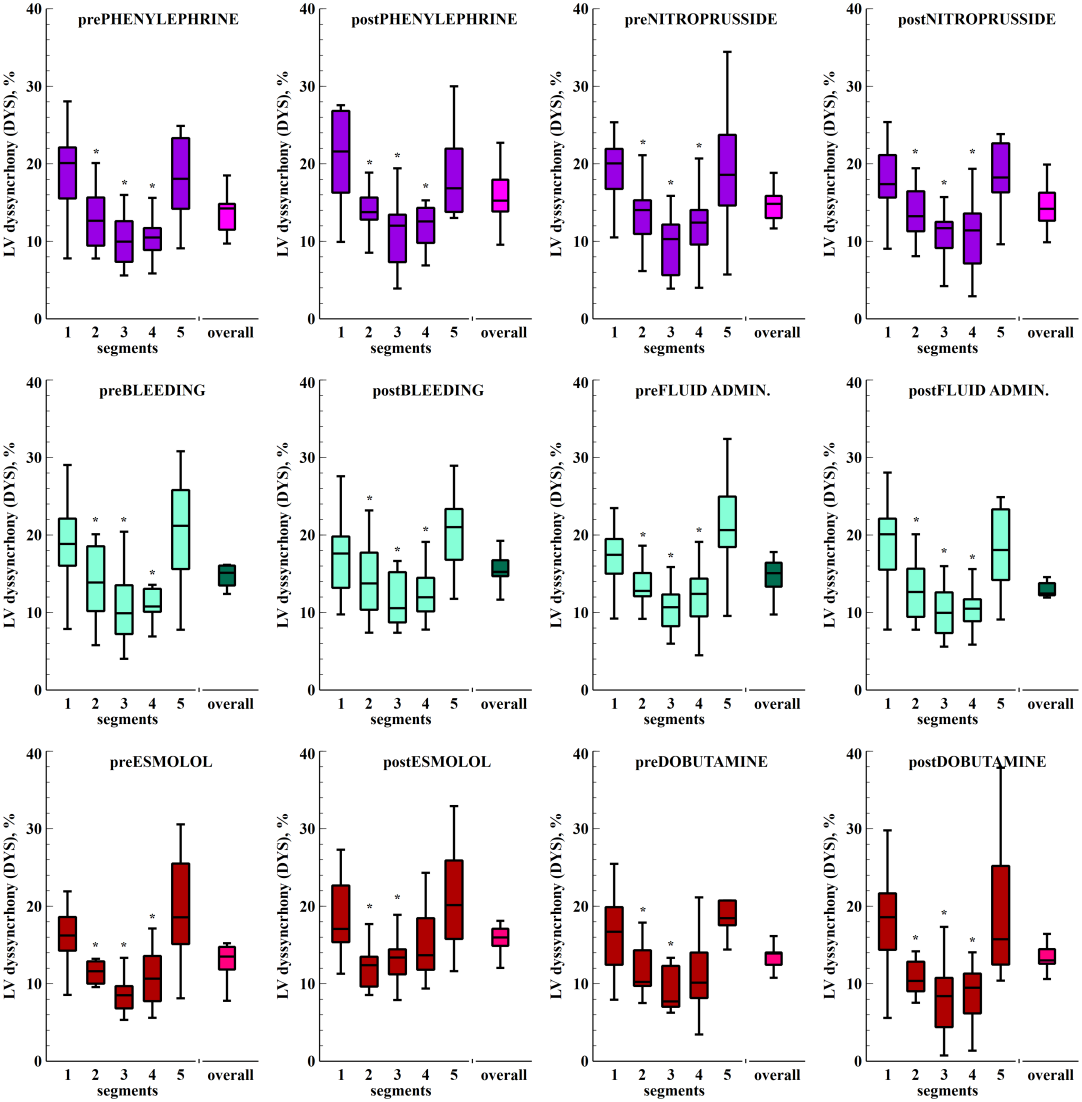
Overall and segmental intraventricular mechanical dyssynchrony during the cardiac cycle (*n* = 13). Analysis of the overall and segmental mechanical dyssynchrony during different stages of the cardiac cycle. Top: The color scale represents the averaged segmental mechanical dyssynchrony during the study. Overall dyssynchrony at each cardiac cycle stage was presented as violin and notched box‐and‐whisker plots. The shaded area defines the probability density (truncated at the 1st and 99th percentile) of the segmental dyssynchrony data (open circles), and the notched box plot the median (middle line) and 25th to 75th interquartile range. The comparison of the overall dyssynchrony during each stage was performed with the Friedman test for repeated measurements, followed by Conover's multiple comparisons test. *p < 0.05 vs previous cardiac stage.

**FIGURE 4:**
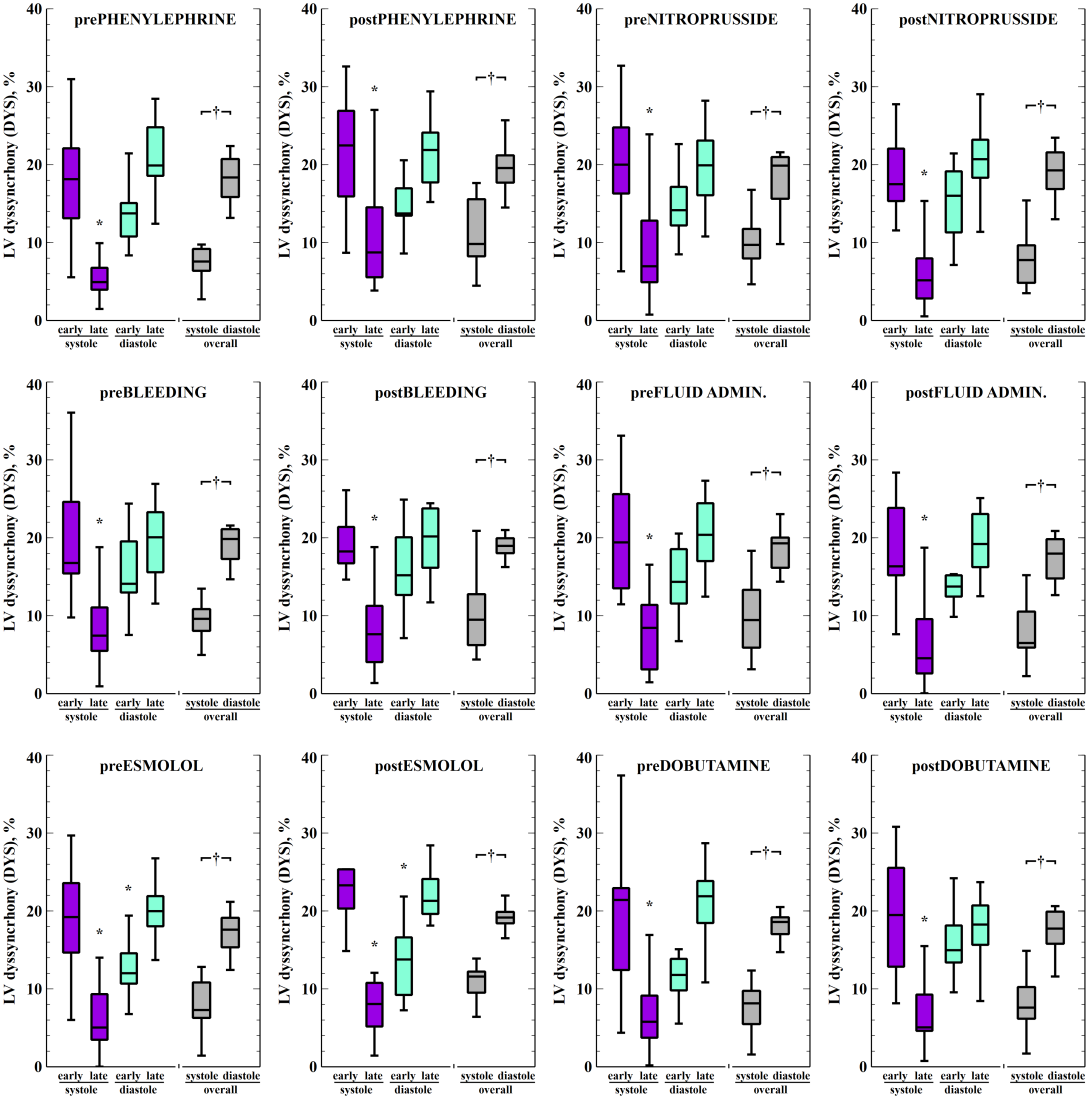
Left ventricular intraventricular mechanical dyssynchrony throughout the cardiac cycle during different experimental interventions (*n* = 13). Box‐and‐whisker plots represent the global mechanical dyssynchrony at different stages of the cardiac cycle. The comparison of different stages was performed with the Friedman test for repeated measurements, followed by Conover's multiple comparisons test. Systolic and diastolic dyssynchrony were compared using a Wilcoxon matched‐pair rank test. *p < 0.05 vs. early systole, ^†^ *p* < 0.05 systole vs. diastole.

**FIGURE 5:**
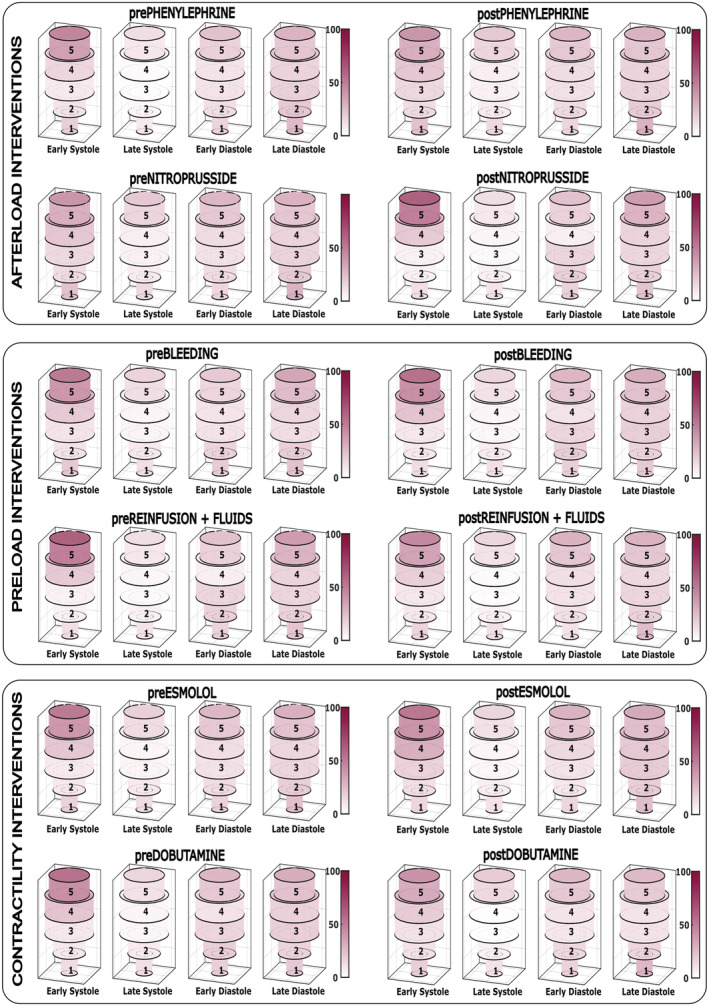
Temporal distribution of the segmental left ventricular intraventricular mechanical dyssynchrony throughout the cardiac cycle during different experimental conditions (*n* = 13). The red color scale denotes the level of mechanical dyssynchrony.

Baseline DYS and IFF were 14 ± 2% and 6 ± 2%, respectively. The temporal distribution of the segmental LVMD throughout the cardiac cycle in baseline conditions is shown in Figure S3. Segmental mechanical dyssynchrony throughout the experiments was higher in basal and apical segments (Figure 3). Overall, DYS decreased during late systole and increased during diastole to similar values as the early systolic level (Figure 4). There was also a base‐to‐apex gradient in DYS more evident during early systole. This pattern was consistent in all experimental stages (Figure 5).

Systolic DYS was only partially related to diastolic DYS (estimate: 0.300; 95% CI: 0.054 to 0.547; *p* = 0.017). The contribution of systolic and diastolic dyssynchrony to the global DYS was 46.5% (95% CI: 45.4% to 47.7%) and 60.2% (95% CI: 58.4% to 62%), respectively. The relationship between global DYS and IFF was strong (*r* ^2^ = 0.848; *p* < 0.001) and described by the exponential function IFF = 0.4839 + 0.001777·DYS + 0.02934·DYS^2^, which implies that ineffective LV volume shifting increases faster at higher DYS levels.

Phenylephrine increased late systolic DYS and IFF, whereas nitroprusside decreased late systolic DYS (Table 1; Table S4). Esmolol increased DYS and IFF more uniformly throughout the cardiac cycle. Dobutamine did not affect overall LVMD, but in the intracardiac cycle analysis, diastolic DYS increased during early diastole and decreased in late diastole (Table S4). Changes in preload were not associated with any significant modification in LV mechanical dyssynchrony.

### 3.4 Relationship between LV mechanical dyssynchrony and LV performance

Systolic DYS and IFF were related to VAC, LV_eff_, and LVEF (Table 2; Figure S4), particularly during late systole (Table S5). The higher the systolic DYS and IFF, the higher the VAC and the lower LV_eff_ and LVEF. A 10% increase in systolic DYS was associated with a 1.1 increase in VAC and a 14% and 15% reduction in LV_eff_ and LVEF, respectively. Increased diastolic DYS and IFF were related to a delayed isovolumetric relaxation (τ), reduced PFR, and higher LV *dP*/*dt*_min_ (minimum value of the first derivative of LV pressure) (Table 3; Table S5). A 10% increase in diastolic DYS was associated with a 6 ms delay in isovolumic relaxation and a reduction of 322 mL s^−1^ in PFR. LVMD was also associated with SV: when global DYS and IFF increased, SV decreased (estimate: −3.230; 95% CI: −4.319 to −2.141, *p* < 0.001; and estimate: −3.456; 95% CI: −4.606 to −2.307, *p* < 0.001, respectively).

**TABLE 2:** Association between intraventricular mechanical dyssynchrony and ventriculo‐arterial coupling, left ventricular mechanical efficiency, and ejection fraction (*n* = 13).

Fixed effects	VAC (Ea/Ees)	LV_eff_	LVEF
Global DYS, %	0.135 (0.096 to 0.174)^†^	−1.781 (−2.359 to −1.202)^†^	−1.896 (−2.450 to −1.342)^†^
Systolic DYS, %	0.111 (0.087 to 0.135)^†^	−1.416 (−1.782 to −1.051)^†^	−1.499 (−1.844 to −1.154)^†^
Diastolic DYS, %	0.010 (−0.028 to 0.048)	−0.167 (−0.743 to 0.409)	−0.189 (−0.733 to 0.356)
Global IFF, %	0.167 (0.128 to 0.206)^†^	−2.218 (−2.801 to −1.636)^†^	−2.328 (−2.884 to −1.773)^†^
Systolic IFF, %	0.144 (0.108 to 0.180)^†^	−1.699 (−2.250 to −1.149)^†^	−1.924 (−2.438 to −1.409)^†^
Diastolic IFF, %	0.013 (−0.028 to 0.055)	−0.444 (−1.078 to −0.189)	−0.304 (−0.897 to 0.289)

*Note*: Data are shown as estimates (95% confidence interval), and statistical significance: ^†^ *p* < 0.001. The relationship between continuous variables were assessed by a generalized linear mixed‐effects model analysis with the restricted maximum likelihood (REML) and the Kenward‐Roger degrees of freedom adjustment, using individual animals as a subject for random factors and experimental stages as repeated measurements. Univariate analysis including as a fixed effect a global variable of mechanical dyssynchrony (global DYS or IFF), and bivariate analysis including systolic and diastolic mechanical dyssynchrony variables (i.e., systolic and diastolic DYS). Estimates reflect the average change in the dependent variable (VAC, LV_eff_, and LVEF) per unit increase of the fixed effect.

Abbreviations: DYS, left intraventricular mechanical dyssynchrony; IFF, internal flow fraction; LVEF, left ventricular ejection fraction; LV_eff_, left ventricular mechanical efficiency; VAC, ventriculo‐arterial coupling (Ea/Ees).

**TABLE 3:** Association between diastolic function variables and intraventricular mechanical dyssynchrony.

Fixed effects	LV stiffness	Tau logistic	PFR	LV *dP*/*dt*_min_
Global DYS	0.000 (−0.001 to 0.000)	0.493 (0.274 to 0.713)^†^	−37.397 (−48.544 to −26.250)^†^	19.253 (1.146 to 37.361)*
Systolic DYS	0.000 (−0.001 to 0.001)	0.062 (−0.082 to 0.205)	−11.447 (−18.910 to −3.984)^†^	−1.408 (−13.524 to 10.708)
Diastolic DYS	−0.001 (−0.002 to 0.000)	0.588 (0.361 to 0.815)^†^	−32.172 (−43.939 to −20.405)^†^	24.826 (5.722 to 43.929)*
Global IFF	0.000 (−0.001 to 0.001)	0.568 (0.338 to 0.797)^†^	−33.185 (−45.470 to −20.900)^†^	11.259 (−8.092 to 30.610)
Systolic IFF	0.001 (0.000 to 0.001)	−0.200 (−0.407 to 0.006)	−2.326 (−13.998 to 9.346)	−7.610 (−26.194 to 10.975)
Diastolic IFF	−0.001 (−0.002 to 0.000)	0.828 (0.590 to 1.066)^†^	−32.791 (−46.224 to −19.358)^†^	21.376 (−0.011 to 42.763)

*Note*: Data are shown as estimates (95% confidence interval), and statistical significance: *p < 0.05, **p < 0.01, ^†^ *p* < 0.001. The relationship between continuous variables was assessed by a generalized linear mixed‐effects model analysis with the restricted maximum likelihood (REML) and the Kenward‐Roger degrees of freedom adjustment, using individual animals as a subject for random factors and experimental stages as repeated measurements. Univariate analysis including as a fixed effect a global variable of mechanical dyssynchrony (DYS or IFF), and bivariate analysis including systolic and diastolic mechanical dyssynchrony variables (i.e., systolic and diastolic DYS). Estimates reflect the average change in the dependent variables (LV stiffness, tau, PFR, and *dP*/*dt*_min_) per unit increase in the fixed effect.

Abbreviations: *dP*/*dt*_min_, minimum rate of LV pressure; DYS, mechanical dyssynchrony; IFF, internal flow fraction; LV, left ventricle; PFR, peak filling rate; tau, time constant of the isovolumetric pressure decay (logistic method).

### 3.5 Relationship between LV mechanical dyssynchrony and atrial systolic function

The contribution of atrial systole to LV preload remained unchanged along the different experimental stages but was associated with significant atrial power and duration changes (Table S6). The contribution of atrial systole to SV was increased with phenylephrine and reduced with nitroprusside. Increased diastolic LVMD was associated with prolonged atrial systole. Furthermore, early diastolic LVMD was related to increased atrial contribution to EDV and SV, and atrial power, whereas late diastolic LVMD was related to reduced atrial power (Table S7).

### 3.6 Factors related to left ventricular mechanical dyssynchrony

We assessed in a univariate and multivariate analysis the potential hemodynamic and electrical factors influencing LVMD (Table 4; Table S8). Reduced heart rate and Ees, increased net LV afterload, and prolonged QRS duration was associated with a high LVMD (Table 4). However, this relationship varied depending on the cardiac cycle phase: while Ees, Ea, and QRS duration were related to systolic DYS, heart rate, and Ea were associated with diastolic LVMD (Table 4).

**TABLE 4:** Multivariate analysis determining the factors associated with global, systolic, and diastolic left intraventricular mechanical dyssynchrony (*n* = 13).

Fixed effects	Global DYS	Systolic DYS	Diastolic DYS
Ees, mmHg/mL	−7.852 (−13.197 to −2.508)**	−14.618 (−22.158 to −7.077)^†^	−2.212 (−7.386 to 2.962)
Ea, mmHg/mL	4.452 (2.796 to 6.109)^†^	7.912 (5.575 to 10.249)^†^	1.950 (0.3.46 to 3.554)*
EDV, mL	−0.002 (−0.015 to 0.010)	0.006 (−0.012 to 0.024)	−0.006 (−0.0158 to 0.007)
Heart rate, bpm	−0.066 (−0.096 to −0.036)^†^	0.018 (−0.029 to 0.064)	−0.109 (−0.138 to −0.080)^†^
QRS duration, ms	0.266 (−0.088 to 0.444)**	0.419 (0.169 to 0.670)^†^	0.097 (−0.076 to 0.269)

Fixed effects	Global IFF	Systolic IFF	Diastolic IFF
Ees, mmHg/mL	−8.531 (−13.407 to −3.655)^†^	−11.378 (−17.173 to −5.584)^†^	−5.140 (−10.432 to 0.152)
Ea, mmHg/mL	5.234 (3.723 to 6.745)^†^	6.747 (4.951 to 8.543)^†^	4.427 (2.743 to 6.110)^†^
EDV, mL	0.002 (−0.009 to 0.014)	0.007 (−0.007 to 0.021)	0.005 (−0.008 to 0.018)
Heart rate, bpm	−0.046 (−0.073 to −0.019)^†^	0.018 (−0.015 to 0.050)	−0.112 (−0.144 to −0.080)^†^
QRS duration, ms	0.201 (0.039 to 0.363)*	0.184 (−0.009 to 0.377)	0.130 (−0.051 to 0.311)

*Note*: Data are shown as estimates (95% confidence interval), and statistical significance: *p < 0.05, **p < 0.01, †*p* ≤ 0.001. The relationship between continuous variables were assessed by a generalized linear mixed‐effects model analysis with the restricted maximum likelihood (REML) and the Kenward‐Roger degrees of freedom adjustment, using individual animals as a subject for random factors and experimental stages as repeated measurements. Estimates reflect the average change in the dependent variable per unit increase in the fixed effects.

Abbreviations: DYS, left intraventricular mechanical dyssynchrony; Ea, effective arterial elastance; EDV, left ventricular end‐diastolic volume; Ees, left ventricular end‐systolic elastance; IFF, internal flow fraction.

## 4 DISCUSSION

This study assessed left intraventricular longitudinal mechanical dyssynchrony using the conductance catheter technique in an intact cardiovascular system under different loading and contractile conditions. Our results are in agreement with early observations by Brutsaert about the role of mechanical dyssynchrony as a physiological modulator of normal LV function (Brutsaert, 1987). We have confirmed that mechanical dyssynchrony is part of the normal cardiac functioning and has a significant relationship with LV performance: increased late systolic LVMD was associated with impaired VAC and a lower LV mechanical efficiency and ejection fraction, whereas increased diastolic dyssynchrony was related to delayed ventricular relaxation, reduced LV filling rate, and increased atrial systolic function. We have also established the main hemodynamic factors associated with LVMD and determined their influence throughout the cardiac cycle: while changes in contractility, afterload, and QRS duration were mainly related to systolic LVMD, heart rate, and afterload were associated with diastolic LVMD. Thus, both hemodynamic factors and intraventricular conduction modulate LVMD.

### 4.1 Occurrence and distribution of LVMD in the intact heart

Local wall motion nonuniformity is part of the normal ventricular function (Badagliacca et al., 2015; Haendchen et al., 1983; Klausner et al., 1982; LeWinter et al., 1975). LeWinter et al. (1975) demonstrated the presence of regional variations in myocardial shortening at different LV areas in normal dog hearts. More recently, Lin et al. (2017), using myocardial motion tracking based on magnetic resonance imaging, demonstrated the existence of an asymmetric distribution of motion patterns of individual myocardial segments in healthy human subjects. These temporal and spatial disparities are related to the intrinsic heterogeneity of the cardiac pump and the physiological nonuniformity of the electromechanical activation and deactivation (Brutsaert, 1987; Glukhov et al., 2010).

In our study, the presence of LVMD was small, affected more basal and apical segments, and was higher during diastole. We have also observed a persistent pattern during early systole consisting in a base‐to‐apex gradient and a significant decrease in mid‐ventricular segments during LV ejection in LVMD. Although we can only speculate about the potential explanation for this pattern, this finding is consistent with the proposed mechanical sequence in the ventricle, which first involves the base of the heart and then extends to the septum and apex (Ballester‐Rodes et al., 2006).

The prevalence of global DYS and IFF at baseline was 14% and 6%, respectively. These values are significantly lower than those reported in patients with chronic heart failure using the conductance catheter for assessing LVMD (Steendijk et al., 2004). Because of the inherent ethical issues related to its invasiveness, there is no information about the incidence of LVMD in healthy subjects using this technique. However, in our study, systolic LVMD was similar to the values previously reported in pigs (Walters et al., 2018, 2020). Kurita et al. (2010) found higher systolic and diastolic DYS levels in dogs (15.9% and 28.7%, respectively). However, these values may differ because of the different volume signal processing or timings used for the cardiac intervals. In a porcine experimental model and using a similar methodology for assessing LVMD, A'Roch et al. (2009) found a systolic DYS of 8% and IFF of 7% in baseline conditions, with a similar segmental distribution and a higher LVMD in apical and basal segments.

Diastolic DYS was higher and only partially related to systolic DYS, which supports the involvement of different mechanisms in systolic and diastolic LVMD. We also observed a consistent decrease in late systolic DYS in all experimental stages, indicating that LVMD is regulated to ensure a highly coordinated and effective ventricular ejection under most physiological conditions. This temporal evolution contrasts with the significant longitudinal and circumferential dyssynchrony observed by Helm et al. (2005) during late systole in an experimental model of heart failure. Moreover, the observed contraction pattern in our study, with a markedly reduced DYS and IFF during late systole, seems to be lost in patients with heart failure, being mechanical dyssynchrony present throughout the cardiac cycle (Kurita et al., 2007).

### 4.2 Relationship between intraventricular mechanical dyssynchrony and LV performance

The coupling between LV and the arterial system is a well‐known determinant of cardiovascular performance and cardiac energetics (Chantler et al., 2008). Our results corroborate that LVMD is associated with VAC and LV mechanical efficiency. Particularly, we have found that increased late systolic dyssynchrony, which takes place mainly during LV ejection, was associated with an impaired LV systolic performance. Although it is unlikely that LVMD affects LV performance by directly reducing intrinsic contractility, the loss of regional coordination during contraction may cause a substantial decrease in pump function (Johnson et al., 2009; Park et al., 1985).

Park et al. (1985) observed that ventricular pacing was associated with a rightward shift of the ESPVR with no change in its slope (Ees), indicating a decline in LV performance that was proportional to the pacing‐induced LVMD. Similarly, Johnson et al. (2009) demonstrated that the pacing‐induced model of LVMD did not affect Ees. They also observed that measured LV PVA underestimated myocardial oxygen consumption during LVMD, which was explained by a rightward shift of the ESPVR as LVMD increased. Although under different experimental conditions than our experiments, these studies demonstrated that, since nonsynchronous ventricular segments are out of phase with global ventricular ejection, they increase heterogeneity during contraction, leading to impaired work transfer to the arterial system, expressed as higher potential energy and reduced SW (Strum & Pinsky, 2000). Furthermore, dyssynchronous contraction is associated with greater myocardial oxygen consumption at any level of contractility, which eventually diminishes myocardial mechanical conversion efficiency (Johnson et al., 2009).

In our study, LVMD remained relatively low throughout the experiment (ranging from 4% to 23%) and lower than the values reported in patients with heart failure (Steendijk et al., 2004, 2006), despite the significant hemodynamic changes induced by our protocol. As the cardiovascular system defends an optimum LV mechanical efficiency over a broad range of loading and contractile conditions (De Tombe et al., 1993; Kubota et al., 1992), it is reasonable to speculate that under physiological conditions, the factors associated with LVMD are aimed at minimizing mechanical dyssynchrony and optimizing LV performance (Brutsaert et al., 1984). On the contrary, mechanical dyssynchrony increases when pathological conditions such as heart failure or LBBB significantly alter those determinants, reducing cardiovascular performance and energy expenditure (Nelson et al., 2000; Steendijk et al., 2006). Moreover, our results agree with the CRT's short‐ and long‐term beneficial hemodynamic effects in patients with heart failure and intraventricular conduction delay (Breithardt et al., 2002; Steendijk et al., 2006). By improving contraction synchrony, CRT results in a more effective ejection and more efficient conversion of the total mechanical energy into SW (Steendijk et al., 2006; Zanon et al., 2009).

### 4.3 LV mechanical dyssynchrony and diastolic function

Diastolic dyssynchrony is a common phenomenon in many pathological conditions, such as LV hypertrophy, arterial hypertension (Chang et al., 2009), diabetes (Kosmala et al., 2004), and coronary artery disease (Lee et al., 2011), and its prevalence in heart failure seems to be at least as frequent as systolic dyssynchrony (Schuster et al., 2005; Yu et al., 2003). Moreover, LVMD may contribute to diastolic dysfunction in these pathological conditions (Lee et al., 2011; Sun et al., 2015).

In our study, increased diastolic mechanical dyssynchrony was associated with impaired ventricular relaxation, reduced LV filling rate, and increased atrial contribution to LV filling. These findings confirm earlier claims about the role of nonuniformity as an independent modulating factor of normal ventricular relaxation (Blaustein & Gaasch, 1983; Brutsaert et al., 1984; Gaasch et al., 1985; Heyndrickx et al., 1988). Gillebert and Lew (1989) and Lew and Rasmussen (1989) demonstrated that increased nonuniformity produced by intracoronary injection of isoproterenol was an independent determinant of the rate of ventricular pressure decay regardless of global loading conditions. Similarly, Zile et al. (1987) observed that pacing‐induced asynchrony slowed the process of isovolumic relaxation and reduced LV filling in open‐chest anesthetized dogs. Aoyagi et al. (1989), using a similar paced‐induced model of dyssynchrony, observed that ventricular wall motion nonuniformity was associated with impaired LV relaxation without significant changes in LV *dP*/*dt*_max_. Therefore, diastolic LVMD modulates changes in diastolic function regardless of the systolic function.

We have also examined the relationship between LVMD and atrial systolic function. Early diastolic LVMD was associated with increased atrial contribution to EDV and SV, atrial power, and prolonged atrial contraction. Therefore, LVMD during passive ventricular filling seems to enhance atrial performance for sustaining cardiac preload. In this regard, Bonow et al. (1988) showed that age‐related diastolic dyssynchrony in normal subjects was associated with reduced global PFR and an increased contribution of atrial systole to global EDV and SV. So, it seems reasonable to speculate that the age‐related increase in diastolic LVMD may contribute to diastolic dysfunction and LV filling abnormalities observed in elderly subjects.

### 4.4 Hemodynamic factors associated with LV mechanical dyssynchrony

We have examined the potential relationship between hemodynamic factors and ventricular depolarization dispersion (QRS duration) on LVMD. During systole, Ees, Ea, and QRS duration were the main factors associated with LVMD, whereas during diastole, only heart rate and Ea were related to LVMD.

In our study, LV afterload played a significant role in systolic and diastolic LVMD. Hayashida et al. (1992) showed that a reduced LV afterload with sodium nitroprusside in patients with dilated cardiomyopathy improved ventricular relaxation and reduced regional asynchrony. Similarly, Miura et al. (1993) found that impaired LV relaxation during increased afterload with angiotensin II was partly due to increased segmental dyssynchrony. Moreover, medical therapy aimed at reducing LV afterload, such as verapamil or enalapril, has also been shown to reduce diastolic LVMD and improve diastolic function (Bonow et al., 1987; Kurita et al., 2010; Kwon et al., 2015). Since nonuniform ventricular wall motion is determined by the myocardium's regional contractility and loading conditions (Brutsaert, 1987; Hayashida et al., 1992), the regional nonuniform wall stress distribution and the interaction between afterload and contractility in the normal ventricle determines then LVMD.

The effects of heart rate in LVMD have been addressed explicitly by Kurita et al. (2007). These authors increased heart rate by atrial pacing and compared systolic DYS and IFF in heart failure patients with LVEF <50% and control subjects referred for elective cardiac catheterization for atypical chest pain. Only patients with heart failure significantly increased systolic LVMD. The authors postulated that the heart rate‐dependent dyssynchrony might contribute to the impaired force‐frequency relation observed in this clinical condition (Eising et al., 1994). In our study, an increase in heart rate was associated with a reduced τ and improved PFR, which is compatible with a frequency‐dependent upregulation of the relaxation processes (Varian & Janssen, 2007).

We have found a significant relationship between LVMD and EDV in the univariate analysis but not in the multivariate one. In an early work, Gillebert and Lew (1989) observed that temporal and regional nonuniformity, assessed by comparing segment lengths in the anterior and posterior ventricular walls, were not affected by volume loading in intact, ejecting ventricle of anesthetized dogs. More recently, in a porcine endotoxic model, A'Roch et al. (2009) demonstrated that both systolic DYS and IFF were unaffected by abrupt preload reductions, as obtained during an IVC maneuver. By contrast, Kim et al. (2011) and Park et al. (2010) showed that preload changes significantly altered systolic LVMD in heart failure patients and nonischemic dilated cardiomyopathy when assessed by tissue imaging echocardiography or by speckle‐tracking radial analysis. Thus, it is likely that the hemodynamic factors, such as preload or heart rate, may affect LVMD differently depending on the patient's physical status (Wachter et al., 2009).

### 4.5 Clinical relevance

Our results showed that LVMD is a dynamic phenomenon that affects LV performance and is influenced by the relative contribution of hemodynamic factors and the electrical intraventricular conduction, expressed as the QRS duration. Consequently, the presence of pathological LVMD could be analyzed from this perspective (Fudim, Dalgaard, et al., 2021; Kass, 2008; Russell et al., 2010). For example, many patients with heart failure but normal ventricular conduction (narrow QRS complex) exhibit an abnormally high LVMD (Bleeker et al., 2005; Cho et al., 2005; Yu et al., 2003). In these patients, hemodynamic factors may be the predominant determinants of LVMD, explaining that altering electrical conduction with CRT is ineffective or even detrimental (Shah et al., 2015). However, correcting hemodynamic factors with medical therapy, such as vasodilators (Kurita et al., 2010; Kwon et al., 2015), could reduce LVMD and have a clinical benefit.

On the contrary, altered electrical and hemodynamic factors could coexist in chronic heart failure patients with abnormal LVMD and delayed intraventricular conduction (Ghio et al., 2004). In this case, CRT has been demonstrated to exert short (Breithardt et al., 2002) and long‐term beneficial effects (Steendijk et al., 2006). However, many patients still fail to respond to this therapy (Kirk & Kass, 2013). Although several factors may contribute to this variability, other possible explanations may account for these patients: first, the impact of hemodynamic factors may prevail over electrical dyssynchrony and persist after CRT, and second, correction of abnormal conduction delay by CRT may predominantly improve systolic over diastolic dyssynchrony (Schuster et al., 2005; Shanks et al., 2010). Consequently, the mere presence of LVMD, independently of the QRS duration, does not guarantee the beneficial effect of CRT (Chung et al., 2008; Shah et al., 2015). Therefore, for an adequate characterization of candidates for CRT, it seems necessary to determine whether electrical dyssynchrony is associated with LVMD and identify its relative contribution (Kirk & Kass, 2013).

### 4.6 Limitations

Several limitations must be acknowledged. First, we assessed the impact of loading and contractile conditions changes in an intact cardiovascular system. The magnitude or direction of these changes in LVMD could be different in pathological conditions, such as in heart failure patients. Second, we did not address the impact on LV performance by directly modifying ventricular electrical conduction. However, previous studies have demonstrated that artificial ventricular pacing significantly increases mechanical dyssynchrony and reduces ventricular mechanical efficiency (Burkhoff et al., 1986; Johnson et al., 2009; Park et al., 1985). So, even if electrical activación was not modified in our study, electrical dyssynchrony has previously been shown to be an independent factor influencing LV systolic performance by increasing dyssynchronous mechanical contraction. Besides, our experimental model was designed to evaluate the impact of acute functional changes in loading and contractile conditions on LVMD, not variations in LV geometry or structure, as they may occur in heart failure or cardiac ischemia (Nagueh, 2008). Third, although the conductance technique for assessing LVMD provides a valuable advantage for analyzing LVMD and LV performance simultaneously, some limitations exist. We assessed LVMD only along the longitudinal axis consisting of stacked cylinders, which is most likely only part of the global LV dyssynchrony. Any anterior to posterior or lateral to intraventricular septum LVMD within cylinders would not be reported. However, prior studies by Gorcsan et al. (1997) showed that, except for pacing‐induced LVMD, such mechanical dyssynchrony is minimal in normal hearts. Moreover, the potential displacement of the conductance catheter during experimental hemodynamic interventions may have been responsible in part of the observed dyssynchrony. However, this limitation, if significant, should also have affected to the temporal and segmental distribution of LVMD values. As LVMD distribution was very consistent during the study, we think that the potential impact of this limitation in our results was minimal.

Fourth, LV torsion, both systolic coiling and diastolic uncoiling, are an essential aspect of LV movement. Lamia et al. (2011) demonstrated that pacing‐induced changes in torsion could profoundly impair LV ejection effectiveness. We did not assess myocardial torsion in this study. However, prior studies have shown that in the absence of heterogeneous myocardial contractile elements or arrhythmias, LV ejection effectiveness due to torsion are minimally altered by changes in afterload, preload, or contractility (Dong et al., 1999).

We have also observed that the filter applied to the LV volume signal significantly affects the dyssynchrony values, particularly during isovolumetric phases, with higher LVMD values when using low‐pass filters with lower cutoff frequencies. Since we used a 25 Hz low‐pass filter in all cases, this limitation did not affect our results. However, the impact of this technical issue should be acknowledged as a potential factor influencing the discrepancies observed in previous and future studies when using the conductance technique. Finally, translating our results to human cardiovascular physiology is inherently limited by the experimental nature of our study and the use of a swine model.

## 5 CONCLUSIONS

In our study, the presence of LVMD was small, affected more basal and apical segments, decreased significantly during late systole, and was higher during diastole. In agreement with previous knowledge, we found that LVMD is an independent physiological variable associated with ventricular contraction and relaxation, although both processes occur in a highly coordinated fashion in normal conditions. An increase in LV intraventricular mechanical dyssynchrony was associated with a decreased mechanical ventricular efficiency and impaired LV relaxation and filling. Both electrical and hemodynamic factors were associated with LV mechanical dyssynchrony.

## AUTHOR CONTRIBUTIONS

Manuel Ignacio Monge Garcia, Michael R. Pinsky, and Maurizio Cecconi contributed to the study conception. Manuel Ignacio Monge Garcia, Zhongping Jian, and Michael R. Pinsky contributed to the study design. Manuel Ignacio Monge Garcia, Zhongping Jian, and Feras Hatib performed experimental research. Manuel Ignacio Monge Garcia, Zhongping Jian, Jos J. Settles, Michael R. Pinsky, and Feras Hatib analyzed and interpreted the data. Manuel Ignacio Monge Garcia, Michael R. Pinsky, Zhongping Jian, Jos J. Settles, and Feras Hatib drafted the manuscript. All authors reviewed it, contributed significantly to its critical review, and approved the final version of the manuscript. All authors ensure the accuracy or integrity of the results of this study and will be accountable for any question related to this work.

## FUNDING INFORMATION

Edwards Lifesciences provided the software, hardware, and animals for the study.

## CONFLICT OF INTEREST STATEMENT

MIMG has been an employee of Edwards Lifesciences and is now a clinical consultant to Edwards Lifesciences. M.R. Pinsky is a consultant to Edwards Lifesciences, LiDCO Ltd., and Cheetah. MC has received honoraria and/or travel expenses from Edwards Lifesciences, LiDCO, Cheetah, Bmeye, Masimo, and Deltex Medical. ZJ, JJS, and FH are Edwards Lifesciences employees.

## ETHICS STATEMENT

The Institutional Animal Care and Use Committee (IACUC) at the Edwards Research Center approved the study protocol (reference number: EW2017‐31), and all procedures were performed following the USDA Animal Welfare Act regulations (AWArs). All animals received humane care in compliance with the US National Institutes of Health *Guide for the Care and Use of Laboratory Animals* (ILAR, NAP, Washington, DC, 2010, 8th edition) (Institute of Laboratory Animal Resources (U.S.). Committee on Care and Use of Laboratory Animals). The Test Facility is accredited by the Association for the Assessment and Accreditation of Laboratory Animal Care, International (AAALACi) and registered with the US Department of Agriculture for conducting animal research. This manuscript adheres to the Animal Research: Reporting of In Vivo Experiments (ARRIVE) guidelines (Percie du Sert et al., 2020).

## Supporting information


Data S1

## Data Availability

Individual and averaged results from analysis used in this article are available in Figshare Repository, at https://doi.org/10.6084/m9.figshare.20178128.
