# Supplementary material for: Relationship between intraventricular mechanical dyssynchrony and left ventricular systolic and diastolic performance: An in vivo experimental study
Source: Physiol Rep. 2023 Feb 17;11(4):e15607. doi: 10.14814/phy2.15607 (PMC9937795; doi:10.14814/phy2.15607)
Supplement: Supplementary file 1 — Data S1 [file PHY2-11-e15607-s001.docx]

**Relationship between intraventricular mechanical dyssynchrony and left ventricular systolic and diastolic performance: an *in vivo* experimental study**

Manuel Ignacio MONGE GARCIA^1^, Zhongping Jian^2^, Feras HATIB^2^, Jos J. SETTLES^2^, Maurizio CECCONI^3^, Michael R. PINSKY^4^

1. Intensive Care Unit, Hospital Universitario SAS Jerez, Jerez de la Frontera, Spain
2. Edwards Lifesciences, Irvine, California, USA.
3. Department Anaesthesia and Intensive Care Units, Humanitas Research Hospital, Humanitas University, Milan, Italy.
4. Department of Critical Care Medicine, University of Pittsburgh School of Medicine, Pittsburgh, USA.

**SUPPLEMENTARY MATERIAL**

| **Table S1. Hemodynamic variables during afterload changes (n=13)** | | | | | | |
| --- | --- | --- | --- | --- | --- | --- |
|  | **Phenylephrine** | |  | **Sodium Nitroprusside** | |  |
|  | Before | After | p value^*^ | Before | After | p value^*^ |
| **Global hemodynamics** | | | | | | |
| CO, l·min^-1^ | 7.8 ± 2.0 | 6.8 ± 1.1 | 0.014 | 7.3 ± 1.5 | 8.5 ± 2.1 | 0.019 |
| SV, ml | 107 ± 21 | 92 ± 13 | 0.009 | 93 ± 19 | 108 ± 20 | <0.001 |
| HR, beats·min^-1^ | 73 ± 10 | 74 ± 9 | 0.391 | 81 ± 20 | 80 ± 21 | 0.577 |
| MAP, mmHg | 77 ± 9 | 110 ± 13 | <0.001 | 82 ± 7 | 53 ± 6 | <0.001 |
| **LV hemodynamics** | | | | | | |
| EDV, ml | 211 ± 48 | 207 ± 46 | 0.270 | 197 ± 40 | 188 ± 42 | 0.154 |
| ESV, ml | 106 ± 46 | 114 ± 41 | 0.098 | 104 ± 40 | 80 ± 43 | 0.006 |
| Ped, mmHg | 11 ± 4 | 15 ± 4 | <0.001 | 9 ± 5 | 5 ± 4 | <0.001 |
| Pes, mmHg | 83 ± 12 | 120 ± 14 | <0.001 | 91 ± 9 | 59 ± 7 | <0.001 |
| Ejection fraction, % | 52 ± 10 | 46 ± 9 | 0.008 | 49 ± 12 | 60 ± 13 | <0.001 |
| dP/dt_max_, mmHg·s^-1^ | 1011 ± 189 | 1218 ± 167 | <0.001 | 1126 ± 231 | 1009 ± 250 | 0.001 |
| **LV diastolic function** | | | | | | |
| LV stiffness (β) | 0.032 ± 0.011 | 0.037 ± 0.013 | 0.237 | 0.029 ± 0.012 | 0.030 ± 0.037 | 0.935 |
| dP/dt_min_, mmHg·s^-1^ | -1354 ± 269 | -1833 ± 307 | <0.001 | -1514 ± 295 | -982 ± 179 | <0.001 |
| PFR, ml·s^-1^ | 870 ± 189 | 652 ± 130 | <0.001 | 836 ± 204 | 1075 ± 246 | <0.001 |
| Tau (τ), ms | 36 ± 5 | 40 ± 5 | <0.001 | 36 ± 5 | 34 ± 3 | 0.039 |
| **Ventriculo-arterial coupling and LV mechanical efficiency** | | | | | | |
| VAC (Ea/Ees) | 2.52 ± 0.58 | 3.22 ± 0.64 | 0.001 | 2.83 ± 0.82 | 1.69 ± 0.65 | <0.001 |
| Ees, mmHg·ml^-1^ | 0.33 ± 0.09 | 0.42 ± 0.08 | <0.001 | 0.38 ± 0.10 | 0.36 ± 0.11 | 0.465 |
| Ea, mmHg·ml^-1^ | 0.81 ± 0.15 | 1.32 ± 0.26 | <0.001 | 1.01 ± 0.23 | 0.62 ± 0.24 | <0.001 |
| LV_eff_, % | 64 ± 10 | 57 ± 10 | <0.001 | 61 ± 11 | 72 ± 14 | <0.001 |
| SW, mmHg·ml | 7048 ± 1341 | 8685 ± 1152 | <0.001 | 7266 ± 1358 | 6670 ± 1472 | 0.006 |
| PE, mmHg·ml | 4412 ± 1803 | 6928 ± 2808 | <0.001 | 4659 ± 1663 | 2883 ± 1917 | <0.001 |
| Values are presented as means ± SD. LV, left ventricle; CO, cardiac output; SV, stroke volume; HR, heart rate; MAP, mean arterial pressure; EDV, left ventricular end-diastolic volume; ESV, left ventricular end-systolic volume; Ped, left ventricular end-diastolic pressure; Pes, left ventricular end-systolic pressure; dP/dt_max_, maximum rate of left ventricular pressure; β, LV chamber stiffness constant obtained from the end-diastolic pressure-volume relationship; dP/dt_min_, the minimum rate of left ventricular pressure; PFR, peak filling rate; τ (tau), time constant of the isovolumetric LV pressure decay (logistic method); VAC, ventriculo-arterial coupling (Ea/Ees); Ees, LV end-systolic elastance; Ea, effective arterial elastance; LV_eff_, left ventricular mechanical efficiency; SW, left ventricular stroke work; PE, left ventricular potential energy.  ^*^ Paired t-test for before and after each experimental intervention. | | | | | | |

| **Table S2. Hemodynamic variables during preload changes (n=13)** | | | | | | |
| --- | --- | --- | --- | --- | --- | --- |
|  | **Bleeding** | |  | **Reinfusion + fluid loading** | |  |
|  | Before | After | p value^*^ | Before | After | p value^*^ |
| **Global hemodynamics** | | | | | | |
| CO, l·min^-1^ | 7.5 ± 1.8 | 7.3 ± 1.8 | 0.607 | 7.4 ± 1.8 | 9.1 ± 2.7 | 0.003 |
| SV, ml | 99 ± 19 | 100 ± 19 | 0.558 | 98 ± 22 | 116 ± 24 | 0.009 |
| HR, beats·min^-1^ | 76 ± 13 | 74 ± 15 | 0.203 | 77 ± 18 | 78 ± 12 | 0.870 |
| MAP, mmHg | 78 ± 12 | 56 ± 8 | <0.001 | 63 ± 8 | 78 ± 12 | 0.004 |
| **LV hemodynamics** | | | | | | |
| EDV, ml | 226 ± 55 | 205 ± 62 | <0.001 | 204 ± 46 | 258 ± 52 | <0.001 |
| ESV, ml | 127 ± 53 | 105 ± 58 | <0.001 | 106 ± 47 | 142 ± 49 | <0.001 |
| Ped, mmHg | 10 ± 3 | 4 ± 4 | <0.001 | 6 ± 4 | 15 ± 3 | <0.001 |
| Pes, mmHg | 83 ± 12 | 64 ± 9 | <0.001 | 73 ± 7 | 84 ± 12 | 0.013 |
| Ejection fraction, % | 46 ± 11 | 52 ± 14 | <0.001 | 50 ± 14 | 46 ± 10 | 0.050 |
| dP/dt_max_, mmHg·s^-1^ | 1020 ± 193 | 873 ± 225 | <0.001 | 975 ± 243 | 1028 ± 186 | 0.270 |
| **LV diastolic function** | | | | | | |
| LV stiffness (β) | 0.029 ± 0.016 | 0.020 ± 0.009 | 0.052 | 0.020 ± 0.008 | 0.037 ± 0.016 | 0.009 |
| dP/dt_min_, mmHg·s^-1^ | -1401 ± 294 | -1080 ± 230 | <0.001 | -1215 ± 260 | -1324 ± 290 | 0.089 |
| PFR, ml·s^-1^ | 835 ± 164 | 892 ± 139 | 0.065 | 884 ± 188 | 981 ± 294 | 0.072 |
| Tau (τ), ms | 36 ± 5 | 35 ± 5 | 0.261 | 36 ± 6 | 35 ± 5 | 0.240 |
| **Ventriculo-arterial coupling and LV mechanical efficiency** | | | | | | |
| VAC (Ea/Ees) | 2.80 ± 0.97 | 2.06 ± 0.80 | 0.005 | 2.27 ± 1.01 | 2.96 ± 0.93 | 0.019 |
| Ees, mmHg·ml^-1^ | 0.34 ± 0.10 | 0.36 ± 0.12 | 0.284 | 0.39 ± 0.14 | 0.27 ± 0.06 | 0.003 |
| Ea, mmHg·ml^-1^ | 0.88 ± 0.27 | 0.66 ± 0.16 | <0.001 | 0.79 ± 0.23 | 0.76 ± 0.23 | 0.745 |
| LV_eff_, % | 58 ± 11 | 66 ± 13 | <0.001 | 65 ± 12 | 58 ± 12 | 0.002 |
| SW, mmHg·ml | 7143 ± 1354 | 6241 ± 1076 | 0.014 | 6790 ± 1154 | 7916 ± 1777 | 0.005 |
| PE, mmHg·ml | 5217 ± 2037 | 3417 ± 2223 | <0.001 | 3849 ± 1777 | 6015 ± 2231 | <0.001 |
| Values are presented as means ± SD. LV, left ventricle; CO, cardiac output; SV, stroke volume; HR, heart rate; MAP, mean arterial pressure; EDV, left ventricular end-diastolic volume; ESV, left ventricular end-systolic volume; Ped, left ventricular end-diastolic pressure; Pes, left ventricular end-systolic pressure; dP/dt_max_, maximum rate of left ventricular pressure; β, LV chamber stiffness constant obtained from the end-diastolic pressure-volume relationship; dP/dt_min_, minimum rate of left ventricular pressure; PFR, peak filling rate; τ (tau), time constant of the isovolumetric LV pressure decay (logistic method); VAC, ventriculo-arterial coupling (Ea/Ees); Ees, LV end-systolic elastance; Ea, effective arterial elastance; LV_eff_, left ventricular mechanical efficiency; SW, left ventricular stroke work; PE, left ventricular potential energy.  ^*^ Paired t-test for before and after each experimental intervention. | | | | | | |

| **Table S3. Hemodynamic variables during contractility changes (n=13)** | | | | | | |
| --- | --- | --- | --- | --- | --- | --- |
|  | **Esmolol** | |  | **Dobutamine** | |  |
|  | Before | After | p value^*^ | Before | After | p value^*^ |
| **Global hemodynamics** | | | | | | |
| CO, l·min^-1^ | 9.4 ± 1.9 | 5.6 ± 1.9 | <0.001 | 8.1 ± 2.1 | 11.5 ± 3.5 | <0.001 |
| SV, ml | 120 ± 21 | 81 ± 25 | <0.001 | 107 ± 20 | 129 ± 31 | <0.001 |
| HR, beats·min^-1^ | 79 ± 11 | 68 ± 8 | <0.001 | 76 ± 13 | 89 ± 16 | <0.001 |
| MAP, mmHg | 71 ± 13 | 50 ± 6 | <0.001 | 70 ± 9 | 84 ± 12 | <0.001 |
| **LV hemodynamics** | | | | | | |
| EDV, ml | 235 ± 50 | 237 ± 53 | 0.692 | 234 ± 46 | 228 ± 37 | 0.152 |
| ESV, ml | 115 ± 50 | 156 ± 47 | <0.001 | 127 ± 45 | 98 ± 41 | <0.001 |
| Ped, mmHg | 13 ± 4 | 10 ± 3 | 0.044 | 13 ± 4 | 14 ± 5 | 0.575 |
| Pes, mmHg | 77 ± 14 | 60 ± 7 | <0.001 | 76 ± 11 | 87 ± 13 | <0.001 |
| Ejection fraction, % | 53 ± 11 | 35 ± 9 | <0.001 | 47 ± 10 | 57 ± 13 | <0.001 |
| dP/dt_max_, mmHg·s^-1^ | 1053 ± 189 | 556 ± 167 | <0.001 | 942 ± 167 | 1767 ± 359 | <0.001 |
| **LV diastolic function** | | | | | | |
| LV stiffness (β) | 0.033 ± 0.017 | 0.044 ± 0.019 | 0.003 | 0.038 ± 0.020 | 0.034 ± 0.023 | 0.467 |
| dP/dt_min_, mmHg·s^-1^ | -1260 ± 287 | -767 ± 157 | <0.001 | -1215 ± 230 | -1566 ± 316 | <0.001 |
| PFR, ml·s^-1^ | 1041 ± 193 | 668 ± 252 | <0.001 | 918 ± 230 | 1158 ± 305 | <0.001 |
| Tau (τ), ms | 34 ± 4 | 40 ± 4 | <0.001 | 35 ± 3 | 32 ± 5 | <0.001 |
| **Ventriculo-arterial coupling and LV mechanical efficiency** | | | | | | |
| VAC (Ea/Ees) | 2.36 ± 0.76 | 2.97 ± 0.76 | 0.018 | 2.83 ± 0.79 | 1.78 ± 0.70 | <0.001 |
| Ees, mmHg·ml^-1^ | 0.29 ± 0.09 | 0.27 ± 0.07 | 0.461 | 0.27 ± 0.07 | 0.43 ± 0.11 | <0.001 |
| Ea, mmHg·ml^-1^ | 0.66 ± 0.18 | 0.79 ± 0.22 | 0.001 | 0.75 ± 0.21 | 0.72 ± 0.24 | 0.303 |
| LV_eff_, % | 65 ± 11 | 47 ± 10 | <0.001 | 59 ± 10 | 71 ± 11 | <0.001 |
| SW, mmHg·ml | 7883 ± 1363 | 4160 ± 1598 | <0.001 | 6870 ± 1239 | 10670 ± 2286 | <0.001 |
| PE, mmHg·ml | 4374 ± 1927 | 4675 ± 1715 | 0.203 | 4870 ± 1931 | 4285 ± 1863 | 0.037 |
| Values are presented as means ± SD. LV, left ventricle; CO, cardiac output; SV, stroke volume; HR, heart rate; MAP, mean arterial pressure; EDV, left ventricular end-diastolic volume; ESV, left ventricular end-systolic volume; Ped, left ventricular end-diastolic pressure; Pes, left ventricular end-systolic pressure; dP/dt_max_, maximum rate of left ventricular pressure; β, LV chamber stiffness constant obtained from the end-diastolic pressure-volume relationship; dP/dt_min_, minimum rate of left ventricular pressure; PFR, peak filling rate; τ (tau), time constant of the isovolumetric LV pressure decay (logistic method); VAC, ventriculo-arterial coupling (Ea/Ees); Ees, LV end-systolic elastance; Ea, effective arterial elastance; LV_eff_, left ventricular mechanical efficiency; SW, left ventricular stroke work; PE, left ventricular potential energy.  ^*^ Paired t-test for before and after each experimental intervention. | | | | | | |

| **Table S4. Left ventricular mechanical dyssynchrony (DYS) during different cardiac cycle intervals (n=13)** | | | | | | | |
| --- | --- | --- | --- | --- | --- | --- | --- |
|  | **Phenylephrine** | |  |  | **Sodium Nitroprusside** | |  |
| LV dyssynchrony, % | Before | After | p-value^*^ |  | Before | After | p value^*^ |
| early systole | 18 ± 7 | 21 ± 8 | 0.053 |  | 19 ± 7 | 18 ± 5 | 0.589 |
| late systole | 6 ± 3 | 10 ± 7 | 0.006 |  | 8 ± 6 | 7 ± 6 | 0.020 |
| early diastole | 14 ± 4 | 15 ± 5 | 0.304 |  | 15 ± 4 | 15 ± 5 | 0.734 |
| late diastole | 21 ± 4 | 21 ± 4 | 0.507 |  | 20 ± 5 | 21 ± 5 | 0.314 |
|  | **Bleeding** | |  |  | **Reinfusion + fluid loading** | |  |
| LV dyssynchrony, % | Before | After | p-value^*^ |  | Before | After | p value^*^ |
| early systole | 20 ± 7 | 20 ± 5 | 0.918 |  | 20 ± 7 | 18 ± 7 | 0.315 |
| late systole | 9 ± 6 | 8 ± 5 | 0.139 |  | 8 ± 5 | 7 ± 6 | 0.580 |
| early diastole | 16 ± 5 | 17 ± 5 | 0.507 |  | 14 ± 4 | 14 ± 5 | 0.808 |
| late diastole | 20 ± 5 | 20 ± 4 | 0.828 |  | 20 ± 4 | 19 ± 4 | 0.386 |
|  | **Esmolol** | |  |  | **Dobutamine** | |  |
| LV dyssynchrony, % | Before | After | p-value^*^ |  | Before | After | p value^*^ |
| early systole | 20 ± 9 | 24 ± 8 | 0.009 |  | 20 ± 10 | 19 ± 8 | 0.663 |
| late systole | 7 ± 4 | 8 ± 3 | 0.156 |  | 7 ± 5 | 7 ± 5 | 0.267 |
| early diastole | 12 ± 3 | 15 ± 5 | 0.023 |  | 12 ± 4 | 15 ± 5 | 0.027 |
| late diastole | 20 ± 4 | 22 ± 4 | 0.035 |  | 21 ± 5 | 17 ± 5 | 0.016 |
| Early systole: from R wave to dP/dt_max_; late systole: from dP/dt_max_ to end-systolic elastance; early diastole: from end-systolic elastance to peak filling rate; late diastole: from peak filling rate to R wave.  * Paired t-test for before and after each experimental intervention. | | | | | | | |

| **Table S5. Multivariate analysis assessing the association between intraventricular mechanical dyssynchrony (DYS) and LV performance and diastolic function indexes (n=13).** | | | |
| --- | --- | --- | --- |
| **Fixed effects** | **VAC (Ea/Ees)** | **LV_eff_** | **LVEF** |
| early systolic DYS | 0.027 (0.011 to 0.043)^†^ | -0.128 (-0.377 to 0.120) | -0.304 (-0.539 to -0.069)^*^ |
| late systolic DYS | 0.085 (0.060 to 0.110)^†^ | -1.128 (-1.510 to -0.746)^†^ | -1.094 (-1.455 to -0.733)^†^ |
| early diastolic DYS | 0.011 (-0.018 to 0.040) | -0.296 (-0.741 to 0.150) | -0.338 (-0.759 to 0.083) |
| late diastolic DYS | -0.003 (-0.024 to 0.029) | -0.037 (-0.445 to 0.372) | 0.007 (-0.379 to 0.393) |
| **Fixed effects** | **Tau logistic** | **PFR** | **LV dP/dt_min_** |
| early systolic DYS | -0.176 (-0.268 to -0.085)^†^ | -1.517 (-6.493 to 3.458) | -4.581 (-12.715 to 3.554) |
| late systolic DYS | 0.149 (0.008 to 0.290)^*^ | -12.406 (-20.052 to -4.760)^**^ | -3.618 (-16.120 to 8.883) |
| early diastolic DYS | 0.210 (0.046 to 0.374)^*^ | -5.605 (-14.521 to 3.310) | 17.172 (2.595 to 31.749)^*^ |
| late diastolic DYS | 0.473 (0.322 to 0.623)^†^ | -23.530 (-31.710 to -15.349)^†^ | 13.066 (-0.309 to 26.440) |
| DYS, left intraventricular mechanical dyssynchrony; IFF, internal flow fraction; VAC, ventriculo-arterial coupling; Ea: effective arterial elastance; Ees: left ventricular end-systolic elastance; LV_eff_, left ventricular mechanical efficiency; LVEF, left ventricular ejection fraction; tau, time constant of the isovolumetric pressure relaxation (logistic method); PFR, peak filling rate; dP/dt_min_, the minimum rate of LV pressure.  Data are shown as estimates (95% confidence interval), and statistical significance: † p ≤ 0.001, ** p < 0.01, * p < 0.05. Generalized linear mixed-effects model analysis with the restricted maximum likelihood (REML) and the Kenward-Roger degrees of freedom adjustment, using individual animals as a subject for random factors and experimental stages as repeated measurements. Estimates reflect the average change in the dependent variable (VAC, LV_eff_, LVEF, tau, PFR, and LV dP/dt_min_) per unit increase of the fixed effects (early systolic DYS, late systolic DYS, early diastolic DYS, and late diastolic DYS). | | | |

| **Table S6. Atrial systolic function assessment (n=13)** | | | | | | | |
| --- | --- | --- | --- | --- | --- | --- | --- |
|  | **Phenylephrine** | |  |  | **Sodium Nitroprusside** | |  |
|  | Before | After | p-value^*^ |  | Before | After | p value^*^ |
| Atrial systole duration, % | 14.1 ± 3.9 | 13.0 ± 3 | 0.099 |  | 13.5 ± 3.5 | 14.7 ± 4.4 | 0.356 |
| Atrial power, W | 0.11 ± 0.04 | 0.21 ± 0.08 | <0.001 |  | 0.13 ± 0.04 | 0.07 ± 0.04 | <0.001 |
| Atrial contribution to EDV, % | 10.6 ± 3.8 | 12.6 ± 4.8 | 0.091 |  | 11.1 ± 3.5 | 11.0 ± 3.8 | 0.906 |
| Atrial contribution to SV, % | 20.3 ± 6.5 | 27.1 ± 9.2 | 0.048 |  | 22.0 ± 6.2 | 17.9 ± 4.1 | 0.032 |
|  | **Bleeding** | |  |  | **Reinfusion + fluid loading** | |  |
|  | Before | After | p-value^*^ |  | Before | After | p value^*^ |
| Atrial systole duration, % | 13.9 ± 3.3 | 18.1 ± 7.6 | 0.049 |  | 15.8 ± 5.4 | 15.7 ± 4.6 | 0.956 |
| Atrial power, W | 0.14 ± 0.10 | 0.07 ± 0.05 | 0.003 |  | 0.09 ± 0.05 | 0.18 ± 0.13 | 0.048 |
| Atrial contribution to EDV, % | 10.6 ± 3.4 | 11.6 ± 4.4 | 0.302 |  | 11.7 ± 3.8 | 11.7 ± 5.3 | 0.992 |
| Atrial contribution to SV, % | 24.0 ± 7.8 | 22.9 ± 9.3 | 0.602 |  | 23.2 ± 4.3 | 23.5 ± 9.5 | 0.929 |
|  | **Esmolol** | |  |  | **Dobutamine** | |  |
|  | Before | After | p-value^*^ |  | Before | After | p value^*^ |
| Atrial systole duration, % | 13.9 ± 3.3 | 16.4 ± 4.5 | 0.110 |  | 15.7 ± 3.6 | 13.4 ± 4.1 | 0.010 |
| Atrial power, W | 0.20 ± 0.16 | 0.07 ± 0.02 | 0.014 |  | 0.16 ± 0.12 | 0.32 ± 0.18 | <0.001 |
| Atrial contribution to EDV, % | 11.5 ± 4.7 | 10.1 ± 3.8 | 0.354 |  | 11.9 ± 4.3 | 13.1 ± 6.2 | 0.377 |
| Atrial contribution to SV, % | 22.8 ± 12.1 | 29.6 ± 10.8 | 0.204 |  | 26.2 ± 10.7 | 23.7 ± 13.7 | 0.225 |
| Atrial systole duration is expressed as the percentage of the cardiac cycle duration.  * Paired t-test for before and after each experimental intervention. | | | | | | | |

| **Table S7. Association between left ventricular mechanical dyssynchrony and left atrial function (n=13)** | | | | | | | | |
| --- | --- | --- | --- | --- | --- | --- | --- | --- |
| **Fixed effects** |  | **Atrial systole duration, %** |  | **Atrial contribution**  **to EDV, %** |  | **Atrial contribution**  **to SV, %** |  | **Atrial power, W** |
| Global DYS |  | 0.221 (0.011 to 0.430)^*^ |  | -0.026 (-0.241 to 0.189) |  | 0.526 (0.059 – 0.994)^*^ |  | -0.006 (-0.013 to -0.001) |
| early systole |  | -0.114 (-0.204 to -0.023)^*^ |  | -0.032 (-0.130 to 0.065) |  | -0.079 (-0.290 – 0.132) |  | 0.002 (-0.001 to 0.005) |
| late systole |  | -0.119 (-0.251 to 0.014) |  | -0.202 (-0.345 to -0.058)^**^ |  | 0.014 (-0.285 – 0.312) |  | -0.003 (-0.007 to 0.002) |
| early diastole |  | 0.175 (0.020 to 0.331)^*^ |  | 0.246 (0.078 to 0.414)^**^ |  | 0.646 (0.287 – 1.001)^†^ |  | 0.008 (0.002 to 0.013)^**^ |
| late diastole |  | 0.398 (0.253 to 0.543)^†^ |  | 0.103 (-0.054 to 0.259) |  | 0.074 /-0.241 – 0.389) |  | -0.012 (-0.017 to -0.007)^†^ |
| Global IFF |  | 0.110 (-0.119 to 0.339) |  | -0.128 (-0.360 to 0.103) |  | 0.194 (-0.215 – 0.602) |  | -0.006 (-0.014 to -0.002) |
| early systole |  | -0.075 (-0.184 to -0.034) |  | -0.010 (-0.107 to 0.127) |  | 0.030 (-0.213 – 0.273) |  | 0.003 (-0.001 to 0.006) |
| late systole |  | -0.175 (-0.559 to 0.208) |  | -0.478 (-0.891 to -0.066)^*^ |  | -0.139 (-0.971 – 0.694) |  | -0.008 (-0.021 to 0.005) |
| early diastole |  | 0.225 (0.085 to 0.365)^**^ |  | 0.231 (0.080 to 0.381)^**^ |  | 0.776 (0.480 – 1.072)^†^ |  | 0.008 (0.003 to 0.013)^**^ |
| late diastole |  | 0.354 (0.210 to 0.499)^†^ |  | 0.037 (-0.119 to 0.193) |  | -0.041 (-0.357 – 0.274) |  | -0.013 (-0.018 to -0.009)^†^ |
| DYS, left ventricular mechanical dyssynchrony; IFF, internal flow fraction. Atrial systole duration is expressed as a percentage of the cardiac cycle duration; atrial contribution to EDV = (atrial systolic volume / end-diastolic volume) × 100; atrial contribution to SV = (atrial systolic volume / left ventricular systolic volume) × 100. Early systole: from R wave to dP/dt_max_; late systole: from dP/dt_max_ to end-systolic elastance; early diastole: from end-systolic elastance to peak filling rate; late diastole: from peak filling rate to R wave.  Data are shown as estimates (95% confidence interval), and statistical significance: ^*^ p < 0.05, ^**^ p < 0.01, ^†^ p ≤ 0.001.  The relationship between continuous variables were assessed by a generalized linear mixed-effects model analysis with the restricted maximum likelihood (REML) and the Kenward-Roger degrees of freedom adjustment, using individual animals as a subject for random factors and experimental stages as repeated measurements. Estimates reflect the average change in the dependent variable per unit increase of the fixed effect. | | | | | | | | |

| **Table S8. Univariate analysis determining the factors associated with global, systolic, and diastolic left intraventricular mechanical dyssynchrony (n=13)** | | | |
| --- | --- | --- | --- |
| **Fixed effect** | **Global DYS** | **Systolic DYS** | **Diastolic DYS** |
| Ees, mmHg∙ml^-1^ | -6.795 (-11.401 to -2.188)^**^ | -14.660 (-21.446 to -7.874)^†^ | -0.459 (-5.010 to 4.091) |
| Ea, mmHg∙ml^-1^ | 3.044 (1.289 to 4.798)^†^ | 5.876 (3.279 to 8.473)^†^ | 1.122 (-0.620 to 2.863) |
| EDV, ml | 0.009 (-0.001 to 0.019) | 0.0029 (0.014 to 0.043)^†^ | -0.005 (-0.015 to 0.005) |
| Heart rate, bpm | -0.050 (-0.083 to -0.016)^**^ | 0.048 (-0.003 to 0.100) | -0.103 (-0.132 to -0.074)^†^ |
| QRS duration, ms | 0.289 (0.124 to 0.455)^†^ | 0.653 (0.414 to 0.892)^†^ | -0.006 (-0.171 to 0.159) |
| **Fixed effect** | **Global IFF** | **Systolic IFF** | **Diastolic IFF** |
| Ees, mmHg∙ml^-1^ | -7.516 (-11.825 to -3.208)^†^ | -10.111 (-15.336 to -4.886)^†^ | -5.015 (-9.698 to -0.332)^*^ |
| Ea, mmHg∙ml^-1^ | 3.709 (2.094 to 5.325)^†^ | 5.161 (3.225 to 7.096)^†^ | 2.400 (0.613 to 4.187)^**^ |
| EDV, ml | 0.012 (0.003 to 0.021)^*^ | 0.019 (0.008 to 0.030)^†^ | 0.006 (-0.004 to 0.016) |
| Heart rate, bpm | -0.028 (-0.061 to 0.004) | 0.039 (0.000 to 0.079)^*^ | -0.084 (-0.116 to -0.052)^†^ |
| QRS duration, ms | 0.287 (0.131 to 0.443)^†^ | 0.386 (0.197 to 0.575)^†^ | 0.191 (0.021 to 0.361)^*^ |
| DYS, left intraventricular mechanical dyssynchrony; IFF, internal flow fraction; Ees: left ventricular end-systolic elastance; Ea: effective arterial elastance; EDV: left ventricular end-diastolic volume.  Data are shown as estimates (95% confidence interval), and statistical significance: * p < 0.05, ^**^ p < 0.01, † p ≤ 0.001.  The relationship between continuous variables were assessed by a generalized linear mixed-effects model analysis with the restricted maximum likelihood (REML) and the Kenward-Roger degrees of freedom adjustment, using individual animals as a subject for random factors and experimental stages as repeated measurements. Estimates reflect the average change in the dependent variable per unit increase of the fixed effects. | | | |

| **Figure S1. Determination of the QRS features in the ECG using the discrete wavelet transform (DWT)** |
| --- |
| 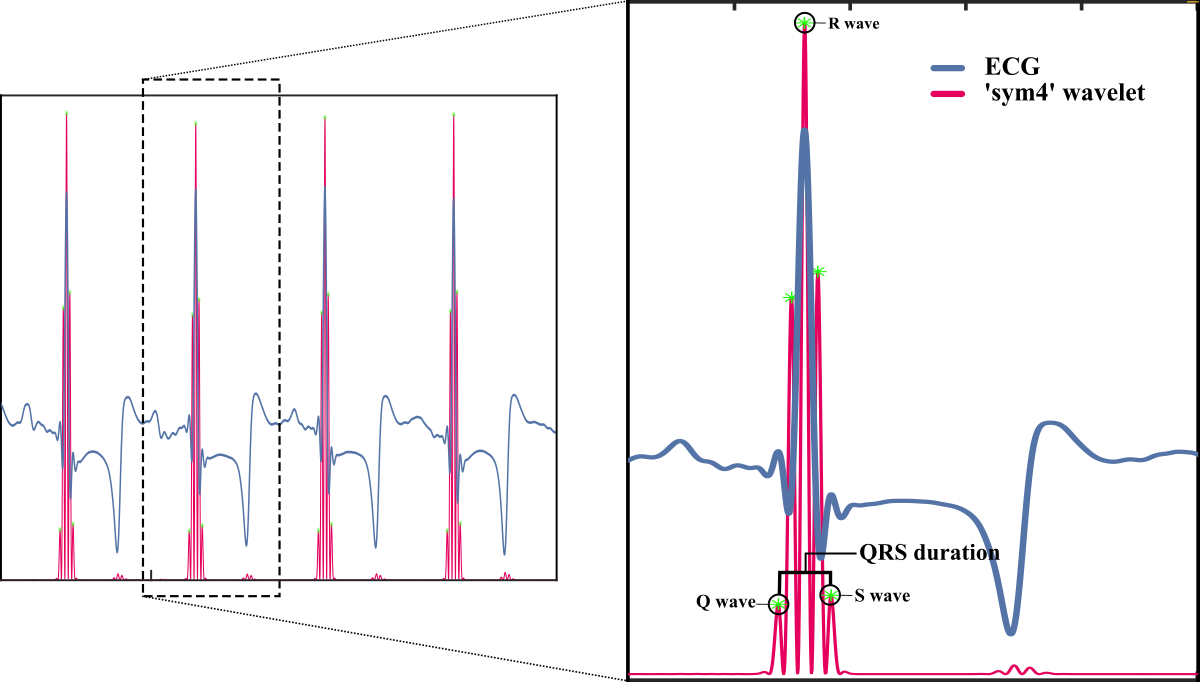 |
| Q wave was identified as the second peak before the maximum point in the wavelet corresponding to the R wave peak. S wave was identified as the second peak after the R wave. More info in Wavelet Analysis of Physiologic Signals, in <https://es.mathworks.com/help/wavelet/ug/wavelet-analysis-of-physiologic-signals.html>. |

| **Figure S2. Atrial systolic function assessment** |
| --- |
| 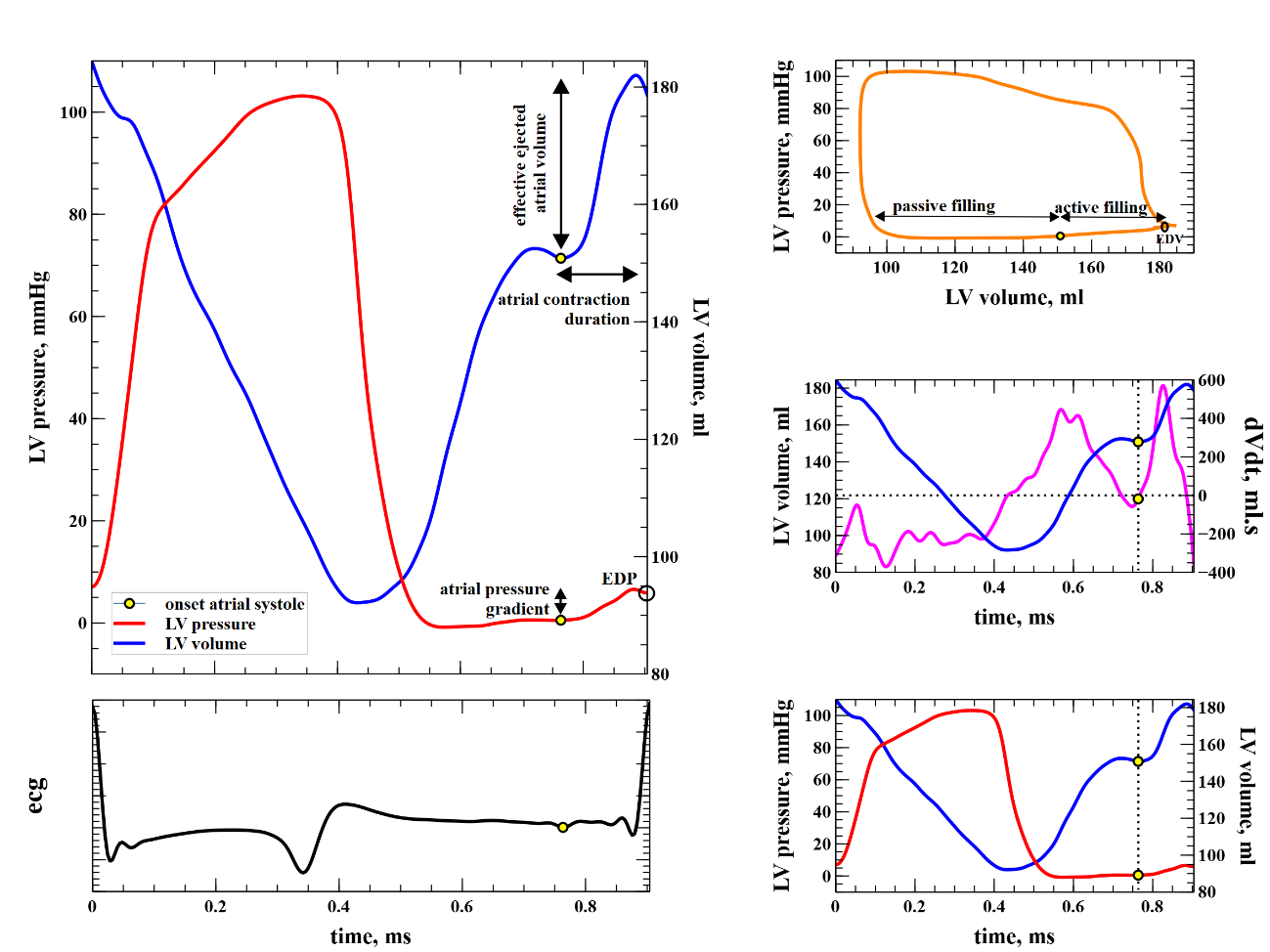 |
| Left ventricular pressure (red) and volume (blue) waveforms, pressure-volume loop (orange), ECG (black) and first derivative of left ventricular volume (dVdt, magenta). The onset of atrial systole is marked with a yellow circle and determined by the zero-crossing of dVdt. |

| **Figure S3. Temporal distribution of the segmental changes in mechanical dyssynchrony throughout the cardiac cycle in baseline conditions** |
| --- |
| 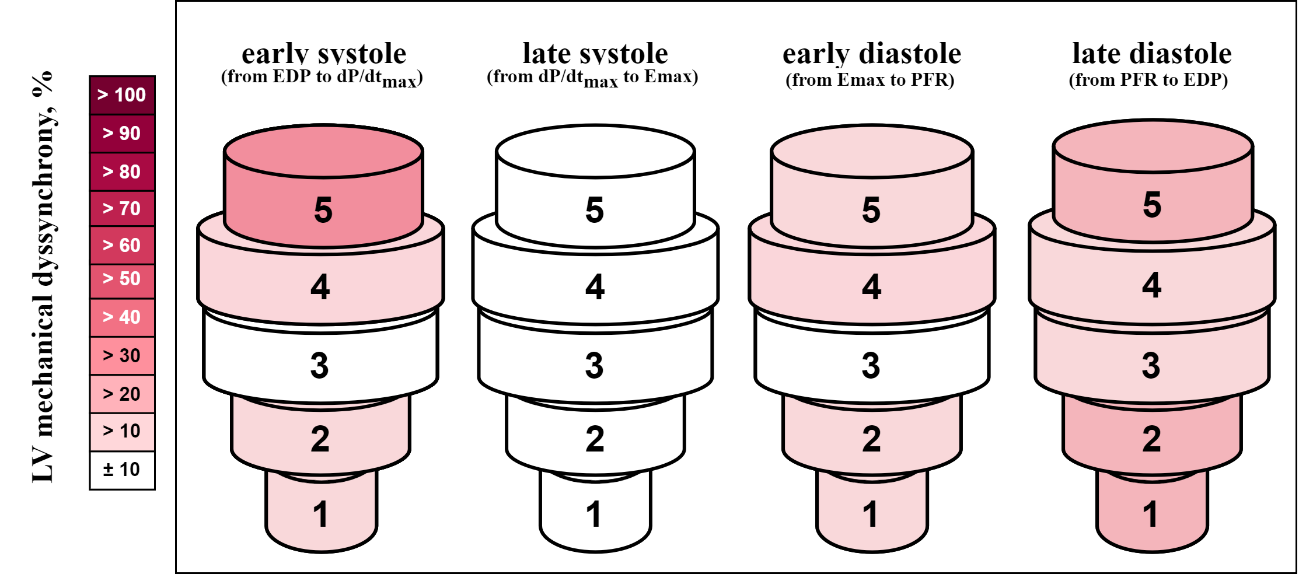 |
| LV: left ventricle; EDP, end-diastolic pressure; dP/dt_max_, maximum rate of left ventricular pressure; Emax, maximum value of left ventricular elastance; PFR, peak filling rate. |

| **Figure S4. Scatter plots for the relationship between systolic and diastolic dyssynchrony indexes with LV systolic and diastolic function.** |
| --- |
| **SYSTOLIC FUNCTION**  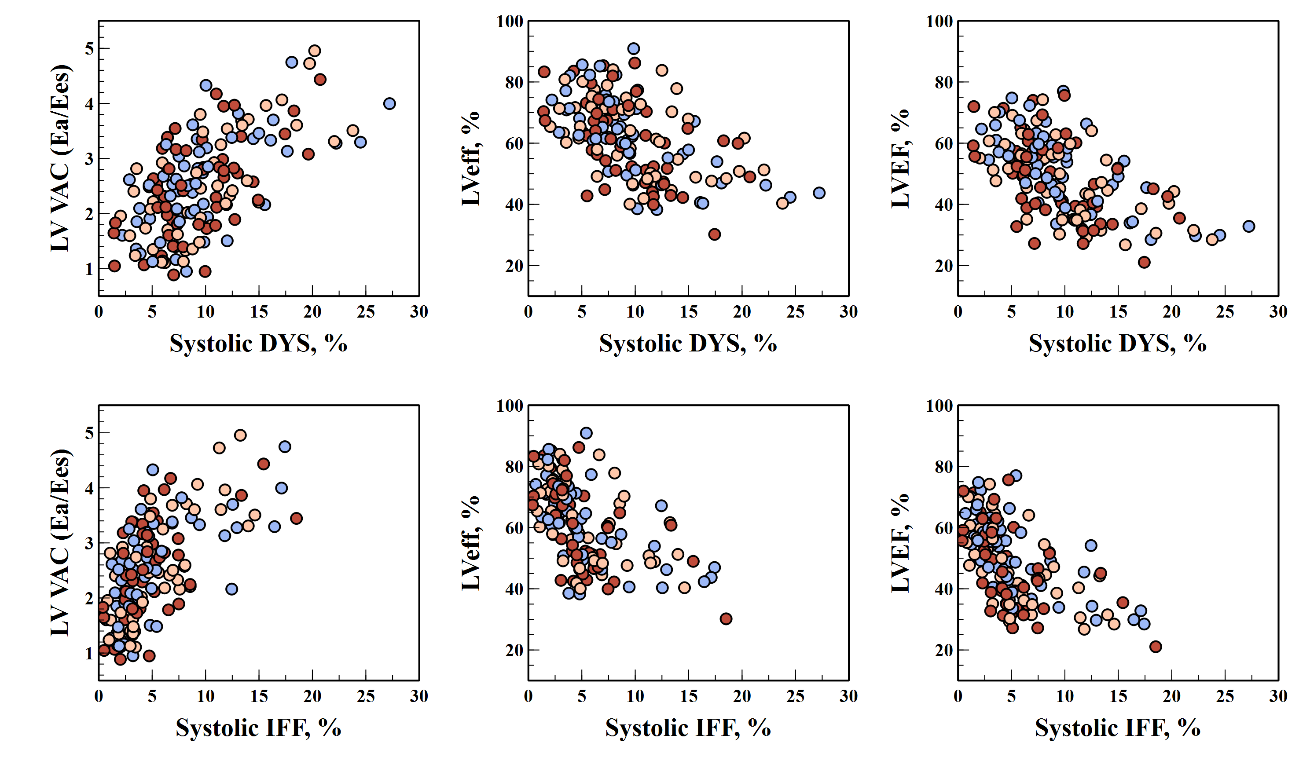 |
| **DIASTOLIC FUNCTION**  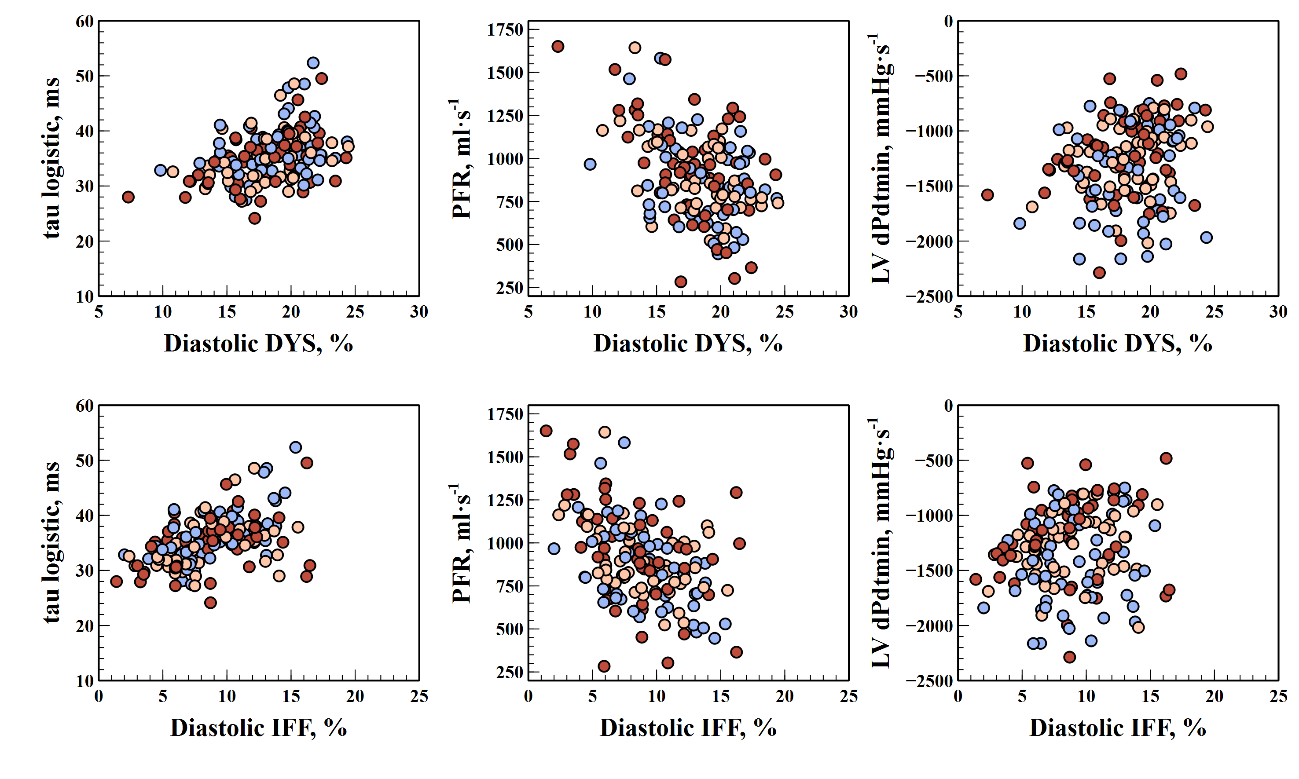 |
| LV: left ventricle; VAC: ventriculo-arterial coupling; LV_eff_: left ventricular mechanical efficiency; LVEF: left ventricular ejection fraction; PFR: peak filling rate; dP/dt_min_: minimum rate of left ventricular pressure; DYS: intraventricular mechanical dyssynchrony; IFF: internal flow fraction.  Colors denote different experimental interventions: light blue for afterload, light red for preload, and dark red for contractility manipulations, respectively. |
